# Trends in care quality in China from 2011 to 2017: An analysis based on the National Specific (Single) Disease Monitoring System

**DOI:** 10.7189/jogh.13.04045

**Published:** 2023-04-28

**Authors:** Chang Yin, Jingkun Li, Wen Meng, Shuang Hou, Dan Liu, Mengyang Liu, Lei Yu, Ruize Guo, Xinhao Han, Meina Liu

**Affiliations:** Department of Biostatistics, School of Public Health, Harbin Medical University, Harbin, China

## Abstract

**Background:**

The Ministry of Health of China conducted a study targeting in single-disease quality control in 2009, aimed to strengthen quality management and improve health care services. This study retrospectively investigated the trends of quality indicators for six monitored diseases 2011-2017 to evaluate the improvement of care quality for the first batch of single-disease.

**Methods:**

We extracted data from the National Specific (Single) Disease Monitoring System for 2011-2017. We focused on six conditions: acute myocardial infarction, heart failure, community-acquired pneumonia, coronary artery bypass graft, hip / knee replacement, and acute ischemic stroke. A total of 56 quality indicators (QIs) were adopted to monitor the quality change and determine the trends in care quality. We also calculated the hospital process composite performance (HPCP) using a denominator-based weighting method for each hospital per year. The estimated annual percentage changes (EAPC) 2011-2017 were calculated at national and regional levels.

**Results:**

The results showed that use of four QIs had significant downward trends, whereas 25 QIs (including reversed indicators) showed significant upward trends from 2011 to 2017. The greatest improvement was observed in CAP-4 (antibiotic treatment within four hours after admission to the hospital for critical pneumonia) in the central region (EAPC = 48.36, 95% CI = 15.92-89.87); while the largest decrease appeared in AIS-1 (thrombolytic therapy within 4.5 hours of symptom onset) in the western region (EAPC = -13.44, 95% CI = -24.98,-0.11). An increased HPCP was observed in four diseases nationwide, but not for acute myocardial infarction and heart failure. However, there were significant differences across regions in the process of care and outcomes, with the performance of Eastern and Western regions showing remarkable advantages compared with the Central region.

**Conclusions:**

We provide evidence for major advancement in care quality in China nationwide. However, the improvement of care in China was unbalanced geographically and should be carefully considered. Future challenges include expanding the coverage of quality monitoring, greater delivery efficiency, and region-balanced health care.

China has shown significant progress in the Healthcare Access and Quality (HAQ) Index 1990-2016; the accelerated pace of progress 2000-2016 confirmed the government’s efforts to achieve universal health coverage [1]. However, evidence also showed large disparities in subnational levels of personal health care access and quality for China [2,3], which emphasized the urgent need to improve both access to and quality of health care across all service areas and for all populations.

Quality of care is the degree to which health services for individuals and populations increase the likelihood of desired health outcomes and are consistent with current professional knowledge [4]. In 2018, the World Health Organization emphasized the importance of a deliberate focus on the quality of health services; this involved providing effective, safe, timely, equitable, integrated, people-centred care, instead of simply ensuring the coexistence of infrastructure, medical supplies, and health care providers [5]. Supplying high-quality care to all individuals in distinct regions is challenging, particularly in a country like China with multiple geographic areas. Regional practices and economic status may impact the care delivery process and outcomes [6]. In 2009, the National Health Commission of the People’s Republic of China launched a quality improvement program. This included building the National Specific (Single) Disease Monitoring System (www.ncis.cn) that aimed to monitor and improve the quality of health care for specific diseases. The first group of diseases entered into the system included acute myocardial infarction (AMI), coronary artery bypass graft (CABG), community-acquired pneumonia (CAP), heart failure (HF), hip / knee replacement (H / K replacement), and acute ischemic stroke (AIS). The National Institute of Hospital Administration issues a quality report (in Chinese) each year in the context of hospitals and medical research institutes nationwide, which aims to promote comprehensive scientific work on quality management [7].

Many studies have focused on temporal trends in care quality of disease, which could provide insight into how health systems are meeting the growing demand for services [8,9]. However, the process quality of health care for specific diseases, including trends and variations, has never been publicly reported in China. This study aimed to quantify and analyse the trends and variations in the quality of health care for the six diseases listed above 2011-2017 at national and regional levels using both single quality indicators (QIs) and the hospital process composite performance (HPCP). The results will provide a macroscopic view of the trends and regionals variations in China health care quality, and a micro insight into QIs.

## METHODS

### Data source

The first group of diseases was selected based on published research and quality reports, such as the Joint Commission’s Annual Report on Quality and Safety [10]. We also considered the prevalence and burden of disease, as well as the national priority areas when identifying the monitoring conditions. Therefore, the data used for this study covered the six diseases that were selected as the first batch of monitoring diseases in the Medical Care Quality Management and Control System for Specific Diseases (AMI, HF, CAP, CABG, H / K replacement, and AIS). Data reporting processes have been published elsewhere [7,11-13]. For additional descriptions, refer to supplementary materials.

The system is an ongoing voluntary, continuous, web-based registration system designed to collect and manage data for QIs. QIs for the system were established by the National Health Commission of the People’s Republic of China in 2009, through a national multidisciplinary board that included clinical and hospital management experts. The process of establishing QIs was based on a systematic literature review, and international and Chinese recommendations. Most QIs were formulated and adapted from the USA [12]. Each QI has an explicit definition. The number of hospitals participating in reporting data in the initial stage of the system was relatively small. However, the number of participating hospitals increased over subsequent years, reaching 634 in 2017; all registered hospitals are secondary or tertiary hospitals from 31 Chinese provinces, autonomous regions, and municipalities, the region the hospitals were sited in was classified as Western, Central, or Eastern according to China Census definitions [14].

Research suggests that the data system covers 74% of tertiary A hospitals in China [15]. Since 2015, data from the Medical Care Quality Management and Control System for Specific Diseases has been released in the “National Medical Service Quality and Safety Report.” Therefore, the hospitals included in this study may be considered representative of “excellent” hospitals in China.

Extraction of case records for the study population was described in the supplementary material. Because the data were fully anonymized and used primarily for hospital care quality management, ethical approval was not required as per China research governance arrangements. Individual patient consent was waived as it was determined that this study did not meet the regulatory definition of human subject research.

### Statistical analysis

The QIs utilization of hospitals annually was calculated based on the definite definition of the indicator denominator (patients who are supposed to satisfy the requirement of that indicator) and the indicator numerator (target patients who meet the requirement of that indicator). The HPCP was calculated at the hospital level for each condition in each year based on the QIs. A denominator-based weighting method was used when calculating weighted HPCP, this method took the percentage of the denominator of each QI to the sum of the denominators of all QIs for the disease as the weight of each QI, which was defined as the sum of eligible patients who actually received care divided by the total care opportunities in this hospital finally [16,17]. The formula is shown in the supplementary material.

We used the estimated annual percentage change (EAPC) to quantify the indicator rates and the HPCP for hospitals trends. The EAPC is a summary and widely used method to show the rate trend over a specified interval [18-20]. Given the seven-year time scope to the reported data, regressions with high-order term may lead to overfitting, a regression line was fitted to the natural logarithm of the rates; that is where y means ln (rate), and x means the calendar year. The EAPC was calculated as and its 95% confidence interval (CI) can also be obtained from the liner regression model. The indicator rates and HPCP were presumed to reflect an increasing trend if the EAPC value and the lower boundary of its 95% CI were both >0. In contrast, the indicator rates and HPCP reflected a decreasing trend if the EAPC value and the upper boundary of its 95% CI were both <0. Other values indicated that trend was stable over time. We calculated the EAPC for each hospital based on years and then summarized it at both the national and regional levels separately.

We also explored the effect of hospital-level covariates on EAPC with generalized additive model which can fit the data under nonlinear conditions. Since the EAPC failed to follow a normal distribution, we used box-cox to transform the EAPC. Although the new variables after the transformation might present difficulties in interpretation, it had an advantage of improving the estimation accuracy of the model and gain more accurate estimation about the factors influencing the trend of EAPC.

The numbers of hospitals differed by year and types of disease. To identify the impact of these changes on interpretation of the data, we performed bootstrap sampling, where 50% of the hospitals involved in reporting were randomly selected each year, and the stability of the findings was displayed visually by drawing the QI trends in the sample hospitals and replicated 1000 times. All statistical analyses were performed using SAS version 9.3, and the figures were developed using GraphPad version 7.0 and R software 4.0.

## RESULTS

**Figure 1:**
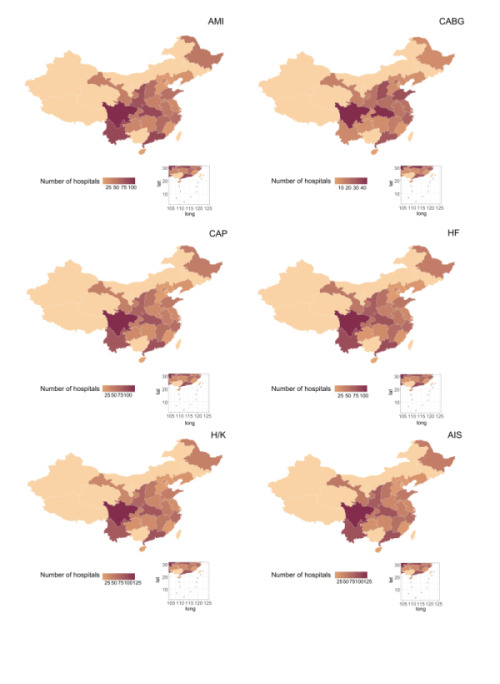
Distribution of reported hospitals throughout China for seven years. The numbers in the figure represent the percentage of the population in the province of China, which is based on the seventh national census. The numbers in the figure represent the percentage of the population in the province. AMI – acute myeloid leukaemia, CABG – coronary artery bypass grafting, CAP – community-acquired pneumonia, HF – heart failure, H / K – hip / knee replacement, AIS – acute ischemic stroke, Qis – quality indicators, HPCP – hospital process composite performance

The definitions of the QIs for each condition are presented in **Table 1** and Tables S1-S6 in the **Online Supplementary Doc**ument. The numbers of hospitals and patients included in this study for each year 2011-2017 are shown in **Table 2**. Data were available up to May 2017, thus the number of hospitals and patients reported in 2017 was relatively low. The geographical distribution of hospitals is shown in **Figure 1**. Although a few autonomous regions did not participate in the data reporting, based on the 2021 national census, the population of the provinces where the participating hospitals are located accounted for already 90% of the national population. Given that not all hospitals were reported consecutively over the seven-year period, we present the characteristics of the registered hospitals grouped by consecutive reporting years (Tables S7-S12 in the **Online Supplementary Document**). Additionally, considering the relatively large number of QIs, we selected representative QIs and showed their EAPCs (95% CI), and the details of other QIs can be found in Tables S13-S18 in the **Online Supplementary Document**.

**Table 1:** Monitoring indicators

Acute myocardial inflation (AMI)
AMI-1. Use of aspirin or clopidogrel immediately on arrival
AMI-2. PCI treatment within 90 min on arrival
AMI-3. Use of beta-blocker immediately on arrival
AMI-4. Use of beta-blocker while in hospital
AMI-5. Use of aspirin / clopidogrel while in hospital
AMI-6. Use of ACEI / ARB while in hospital
AMI-7. Use of statins while in hospital
AMI-8. Continue to use beta-blocker after discharge
AMI-9. Continue to use aspirin / clopidogrel after discharge
AMI-10. Continue to use ACEI / ARB after discharge
AMI-11. Continue to use statins after discharge
AMI-12. Smoking cessation, health counselling, and secondary prevention education
AMI-13. In-hospital mortality
**Heart Failure (HF)**
HF-1. Assessment of left ventricular function
HF-2. Use of ACEI / ARB immediately on arrival
HF-3. Use of beta-blocker as early as possible on arrival
HF-4. Use of diuretics and potassium while in hospital
HF-5. Use of ACEI / ARB while in hospital
HF-6. Use of beta-blocker while in hospital
HF-7. Use of aldosterone antagonists while in hospital
HF-8. Continue to use diuretics and potassium after discharge
HF-9. Continue to use ACEI / ARB after discharge
HF-10. Continue to use beta-blocker after discharge
HF-11. Continue to use aldosterone antagonists after discharge
HF-12. Health education
HF-13. In-hospital mortality
**Coronary artery bypass grafting (CABG)**
CABG-1. Use of the internal mammary artery (the first vessel graft)
CABG-2. Reasonable selection of prophylactic antibiotics
CABG-3. Use of preventive antibiotics within one hour before the operation
CABG-4. Stop using antibiotics within 72 h after the operation
CABG-5. Health education
CABG-6. In-hospital mortality
**Community-acquired pneumonia (CAP)**
CAP-1. Aetiology diagnosis for non-critical pneumonia
CAP-2. Aetiology diagnosis for critical severe pneumonia
CAP-3. Antibiotic treatment within four hours after admission to the hospital for non-critical pneumonia
CAP-4. Antibiotic treatment within four hours after admission to the hospital for critical pneumonia
CAP-5. Selection of proper antibiotics for non-critical patients conforms to the guidelines
CAP-6. Selection of proper antibiotics for critical patients conforms to the guidelines
CAP-7. Discharge within 14 d
CAP-8. In-hospital mortality
**Hip / knee replacement (H / K replacement)**
H/K-1. Selection of preventive antibiotics in line with specifications
H/K-2. Use of prophylactic antibiotic within one hour before the operation
H/K-3. Use of additional antibiotics for operations that last for more than three hours
H/K-4. Application of preventive prophylactic anticoagulants within 24 h
H/K-5. Unilateral surgical blood transfusion more than 400 ml / bilateral 800 ml (reversed indicator)
H/K-6. Health education
H/K-7. Discharge within 21 d
**Acute cerebral infarction (AIS)**
AIS-1. Thrombolytic therapy within 4.5 h of symptom onset
AIS-2. Anticoagulation in patients with atrial fibrillation
AIS-3. Use of aspirin or clopidogrel within 48 h after admission to hospital
AIS-4. Dysphagia assessment
AIS-5. Preventive of deep vein thrombosis (drug therapy)
AIS-6. Preventive of deep vein thrombosis (physical therapy)
AIS-7. Preventive of deep vein thrombosis (rehabilitation therapy)
AIS-8. Health education
AIS-9. In-hospital mortality

PCI – percutaneous transluminal coronary intervention, ACEI – angiotensin-converting enzyme inhibitors, ARB – angiotensin receptor blockers, min – minutes, d – days, h – hours, ml – millilitres

**Table 2:** The number of hospitals and patients included in this study

Year	AMI		HF		CAP		CABG		H/K		AIS	
H	P	H	P	H	P	H	P	H	P	H	P
2011	284	28 542	219	19 300	214	31 660	55	6818	235	24 627	295	71 553
2012	323	34 088	261	30 237	266	37 725	75	10 327	280	32 325	348	95 528
2013	350	36 177	316	37 604	291	43 763	84	11 935	309	41 185	381	106 297
2014	274	26 598	265	31 457	241	37 113	52	8163	273	31 433	320	82 930
2015	276	28 452	251	28 679	209	39 409	50	7762	228	32 714	297	83 022
2016	282	27 322	240	31 973	243	43 213	45	4549	234	30 023	305	90 697
2017	192	10 572	180	15 746	162	19 125	18	1408	141	10 676	225	38 033

AMI – acute myeloid leukaemia, CABG – coronary artery bypass grafting, CAP – community-acquired pneumonia, HF – heart failure, H / K – hip / knee replacement, AIS – acute ischemic stroke, H – the number of hospitals, P – the number of patients

### Acute myocardial infarction (AMI)

**Figure 2:**
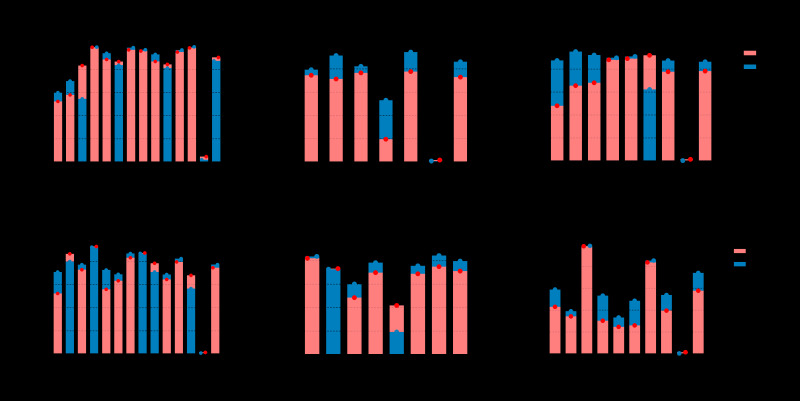
Performance of quality indicators and hospital process composite performance in 2011 and 2017. EAPC – estimated annual percentage change, AMI – acute myeloid leukaemia, CABG – coronary artery bypass grafting, CAP – community-acquired pneumonia, HF – heart failure, H / K – hip / knee replacement, AIS – acute ischemic stroke, Qis – quality indicators, HPCP – hospital process composite performance

**Figure 3:**
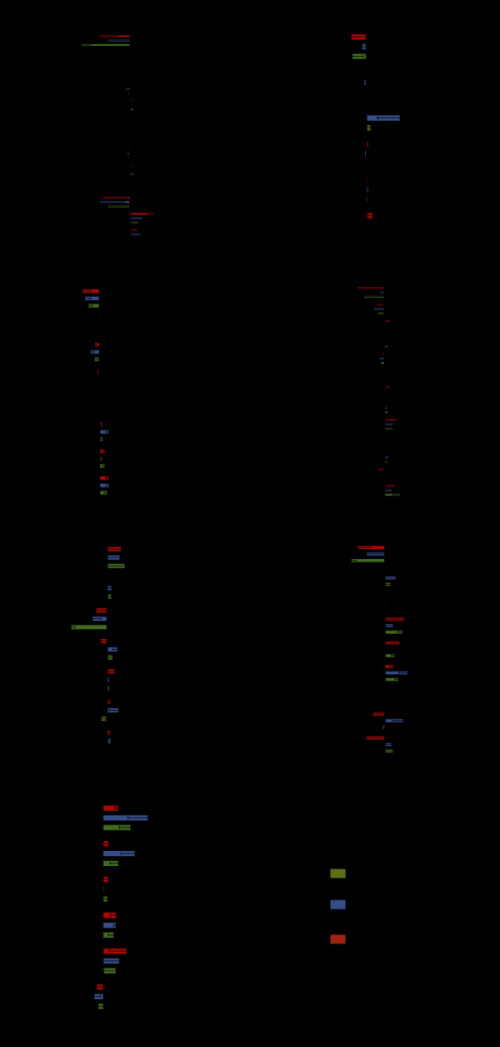
The trends of Specific (Single) Disease based on estimated annual percentage change. x-axis represents EAPC – estimated annual percentage change, AMI – acute myeloid leukaemia, CABG – coronary artery bypass grafting, CAP – community-acquired pneumonia, HF – heart failure, H / K – hip / knee replacement, AIS – acute ischemic stroke, Qis – quality indicators; HPCP – hospital process composite performance

Five of 12 QIs for AMI showed significantly increasing trends, two (including hospital mortality, which was an inverse indicator) displayed a significant decrease, and five did not show significant trends (**Figure 2**, **Figure 3**, and Table S13 in the **Online Supplementary Document**).

The largest gains in QIs occurred in PCI within 90 minutes (AMI-2) annually (EAPC = 4.21, 95% CI = 1.37-7.13), followed by the use of beta-blockers while in hospital (AMI-5, EAPC = 1.23, 95% CI = 0.67-1.87) and continuing use of beta-blockers after discharge (AMI-9, EAPC = 1.24, 95% CI = 0.70-1.79). Use of statins while in hospital (AMI-7) and after discharge (AMI-11) increased only moderately 2011-2017. The hospital mortality rate (AMI-13, EAPC = -14.27, 95% CI = -17.20,-11.24) showed a significant decrease. In addition, immediate use of beta-blockers on arrival (AMI-3, EAPC = -9.00, 95% CI = -16.37,-0.97) showed a decreasing trend. Regionally, the Eastern region performed best, and had the largest decline in in-hospital mortality.

**Figure 4:**
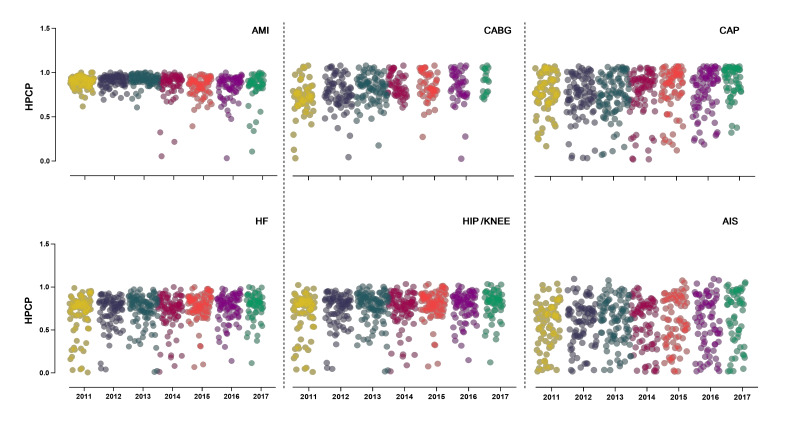
The composite score of hospitals from 2011 to 2017. AMI – acute myocardial inflation, HF – heart failure, CABG – coronary artery bypass grafting, CAP – community-acquired pneumonia, H / K – hip / knee replacement, AIS – acute cerebral infarction, HPCP – hospital process composite performance

Analysis of the HPCP showed the overall trend of quality of AMI care was steady 2011-2017, but there was a downward trend in the Central region (**Figure 3**, **Figure 4**, Table S13 in the **Online Supplementary Document**).

### Coronary artery bypass graft (CABG)

Six QIs were included as core monitoring QIs for CABG, five of which showed little difference in 2011-2017 nationwide. However, there was a notable downward trend in hospital death (CABG-6, EAPC = -16.91, 95% CI = -28.28,-3.74) (**Figure 2** and **Figure 3**). Regionally, a reasonable selection of prophylactic antibiotics (CABG-2) and stopping using antibiotics within 72 hours after the operation (CABG-4) both increased 2011-2017 in the Central region. The latter showed a significant increase, particularly with an EAPC of about half.

The HPCP for CABG showed a steadily increasing trend (EAPC = 2.23, 95% CI = 0.83-3.64), especially in the Western region, but the quartile range widened 2011-2017 (**Figure 3**, **Figure 4**, Table S14 in the **Online Supplementary Document**).

### Community-acquired pneumonia (CAP)

Five of eight QIs for CAP showed significantly increasing trends, and the remaining two QIs had significant decreases (**Figure 2** and **Figure 3**). The highest EAPC was found in the aetiology diagnosis for non-critical pneumonia (CAP-1, EAPC = 12.45, 95% CI = 7.76-17.35), followed by critical pneumonia (CAP-2). The rates of both QIs rose markedly over time, and the gap between the rate of CAP-1 and CAP-2 had been narrowed to 7.74% in 2017. Antibiotic treatment within four hours after hospital admission for non-critical pneumonia (CAP-3) had improved markedly, with a growth of 24.08% 2011-2017. The greatest decrease was observed in hospital deaths (CAP-8, EAPC = -19.57, 95% CI = -24.22,-14.63). Only CAP-5 showed an upward trend in the Eastern region and a downward trend in the Western region.

The HPCP showed CAP increased from 72.56% in 2011 to 81.54% in 2017 at a slow but steady pace (EAPC = 1.69, 95% CI = 1.44-1.95) at both national and regional levels (**Figure 3**, **Figure 4**, Table S15 in the **Online Supplementary Document**).

### Heart failure (HF)

Nationwide, four of 12 QIs for HF had increased rates across the seven years, with the most pronounced increase in the assessment of left ventricular function before treatment from 52.54% in 2011 to 70.99% in 2017 (HF-1, EAPC = 5.68, 95% CI = 4.19-7.18). This was followed by use of beta-blockers while in hospital (HF-6, EAPC = 1.39, 95% CI = 0.95-1.82), use of beta-blockers immediately on arrival (HF-3, EAPC = 1.21, 95% CI = 0.16-2.27) and continuing use of beta-blockers after discharge (HF-10, EAPC = 1.11, 95% CI = 0.50-1.72). The EAPC for hospital death (HF-13) was -8.43, but its CI included 0. The other QIs remained relatively stable nationally over time. At the regional level, among the four QIs (HF-1, HF-3, HF-6, and HF-10) that continued to increase, only the Eastern region showed a significant upward trend (**Figure 2**, **Figure 3**, Table S16 in the **Online Supplementary Document**).

The HPCP showed care quality for HF remained stable over time at both national and regional levels, and the quartile range also narrowed over time (**Figure 3**, **Figure 4**, Table S16 in the **Online Supplementary Document**).

### Hip / knee (H / K) replacement

Nationally, two out of seven QIs for H / K replacement showed significantly increasing trends, one had a significant decrease (**Figure 2** and **Figure 3**). Pronounced increases were detected in application of preventive prophylactic anticoagulants within 24 hours (H / K-4, EAPC = 1.87, 95% CI = 0.16-3.60) and health education (H / K-6, EAPC = 1.68, 95% CI = 0.31-3.06). Unilateral surgical blood transfusion more than 400 ml / bilateral 800 ml (H/K-5) was a reversed indicator whose lower rate indicates better quality. The rate dropped from 41.92% in 2011 to 18.97% in 2017, with a significant and steady downward trend (EAPC = -12.60, 95% CI = -15.53,-9.57). However, at the regional level, these changed dramatically. In the Central region, except for H / K-4, selection of preventive antibiotics in line with specifications (H / K-1) and use of prophylactic antibiotics within one hour before operation (H / K-2) also rose steadily. Only the Eastern and Central regions showed a downward trend in (H / K-5).

The HPCP showed the overall quality of H / K replacement improved from 72.51% in 2011 to 85.19% in 2017 (EAPC = 2.04, 95% CI = 1.49-2.61) nationwide, and the quartile range among hospitals tended to narrow. Regionally, there was an increase in the Eastern and Central regions annually, but a non-significant change was found in the Western region (**Figure 3**, **Figure 4**, Table S17 in the **Online Supplementary Document**).

### Acute ischemic stroke (AIS)

Across the country, four out of nine QIs showed rapid increases, one QI presented a markedly downward trend (**Figure 2**, **Figure 3**, Table S18 in the **Online Supplementary Document**). The most pronounced increase was detected in prevention of deep vein thrombosis, with a drastic increase of 23.63% (physical therapy, AIS-6, EAPC = 11.77, 95% CI = 7.44-16.28). Relatively high increases were observed in dysphagia assessment (AIS-4, EAPC = 9.78, 95% CI = 8.88-10.68) and prevention of deep vein thrombosis (drug therapy, AIS-5, EAPC = 5.90, 95% CI = 3.42-8.43). Moreover, the largest decrease was found in hospital death (AIS-9, EAPC = -19.70, 95% CI = -25.79,-13.11). Regionally, there were upward trends in thrombolytic therapy within 4.5 hours of symptom onset to hospital (AIS-1) in the Eastern region and anticoagulation in patients with atrial fibrillation (AIS-2) in the Central region. It is worth noting that improvement in hospital death (AIS-9) was not found in the Central region.

The HPCP showed care quality for AIS increased steadily from 56.97% in 2011 to 69.56% in 2017 at both the national and regional levels, which was the highest among the six diseases (EAPC = 3.91, 95% CI = 3.20-4.62 (**Figure 3**, **Figure 4**, Table S18 in the **Online Supplementary Document**).

### Association of estimated annual percentage changes (EAPC) and hospital covariates

**Figure 5:**
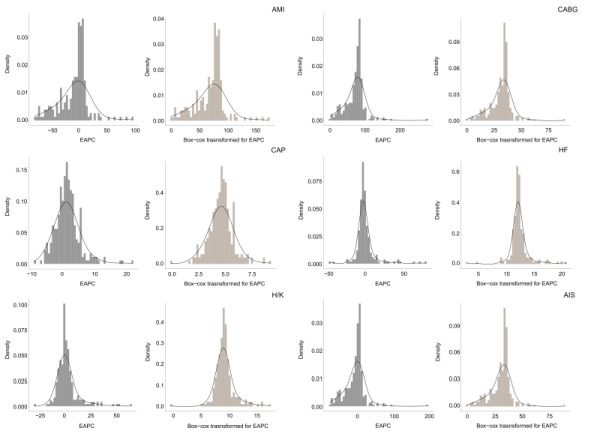
Histogram of estimated annual percentage change (EAPC) frequency distribution before and after box-cox transformation. AMI – acute myocardial inflation, HF – heart failure, CABG – coronary artery bypass grafting, CAP – community-acquired pneumonia, H / K – hip / knee replacement, AIS – acute cerebral infarction, HPCP – hospital process composite performance

The comparison of EAPC before and after transformation is shown in **Figure 5**. In view of the potential association between hospital level covariates and EAPC of HPCP, we included the form of ownership, type of institution, hospital level, hospital class, commissioning hospital, university-affiliated hospital, hospital affiliation, and number of health care quality professionals into stepwise multivariate linear model to examine the covariates influencing the EAPC. We showed the covariates with p less than 0.1 (**Table 3**). The results indicated that tertiary grade-A hospitals, university-affiliated hospitals impacted positively on EAPC. Provincial and municipal hospitals had higher EAPC compared to county hospitals. we also found that hospitals with more medical quality specialized staff were beneficial to improve EPAC, In the analysis of the AMI, no covariates entered the model.

**Table 3:** Impact of hospital-level covariates on EAPC of HPCP

Parameter	CABG		CAP		HF		H / K		AIS	
β (SE)	*P*	β (SE)	*P*	β (SE)	*P*	β (SE)	*P*	β (SE)	*P*
Hospital level (Reference = secondary)	-	-	-	-	-	-	-	-	12.81 (6.10)	0.039
Hospital grade (Reference = grade B)	-	-	-	-	-	-	0.65 (0.39)	0.090	13.09 (6.04)	0.033
Affiliated University Hospital (Reference = not affiliated)	13.61 (3.97)	<0.0001	-	-	-	-	-	-	-	-
Subordination (Reference = county)	9.40 (2.92)	0.002	-	-	0.84 (0.26)	0.001	0.41 (0.24)	0.098	-	-
Number of medical quality specialized staff	-	-	0.60 (0.17)	<0.0001	0.77 (0.29)	0.019	-	-	4.99 (2.24)	0.028
R square	0.140		0.077		0.129		0.047		0.123	

AMI – acute myeloid leukaemia, CABG – coronary artery bypass grafting, CAP – community-acquired pneumonia, HF – heart failure, H / K – hip / knee replacement, AIS – acute ischemic stroke, H – the number of hospitals, P – the number of patients, β – estimated regression coefficients, SE – standard error

## DISCUSSION

China has witnessed marked national progress in the care quality for specific diseases 2011-2017. The National Health Commission announced that more than 40 national-level and 1400 provincial-level quality control centres had been established in China until 2020, and broadened the range of QIs involved in monitoring for continuous improvement of medical quality [21]. These measures delivered achievements of care quality to the public in China, which also can be observed from the trends in the compliance rates for QIs and the HPCP.

To our knowledge, we present the most extensive study on the care quality for specific diseases in China. We found that in four of the six studied diseases (the first batch entered in the monitoring system), the HPCP for process indicators showed steadily increasing progress, especially in the Eastern region. Examination of the performance of single indicators showed that most had upward trends, and only a few had significant downward trends. Given the different numbers of hospitals in different years and diseases, we estimated the trends for monitoring indicators using the bootstrap method. This showed the trend of most indicators was relatively stable and approximately linear, the trend of 1000 samples were integrated into a cluster, with few indicators showing two or more trends (Figures S1-S6 in the **Online Supplementary Document**).

There are regional inequalities in Chinese economic development, with the Eastern region performing more prominently and economically developed regions tending to have relatively high quality and quantity of health workers [22,23]. This factor may affect the balance of health care institutions and services. With the general improvement of care quality across China, we also found significant geographic variation among QIs and HPCPs. The performance of the Eastern region was impressive, with 22 indicators on the rise, and five of the six reversed indicators (including hospital mortality and bleeding) showing downward trends. The Central and Western regions lagged compared with the east. Twelve indicators in the Central region displayed upward trends, and three indicators displayed downward trends. There were rises in 18 indicators in the Western region. Strikingly, some indicators with the greatest increases were in the Western region. This finding was consistent with Zhong et al. [6] which might partly be attributable to the Western Development Program. This program was launched by the Chinese government in 2000 to uphold economic growth and health care in the Western region; the region received 86.3 billion RMB (approximately 12.8 billion US dollars) funding for health, accounting for 46.7% of Chinese public health funds 2001-2009 [24].

The progress on the process quality of specific diseases from 2011 to 2017 showed the effectiveness of the series of actions related to quality improvement implemented by the government. China has made substantial investments in health care and has issued several policy documents highlighting the importance of care quality. Examples include the Measures for the Management of Medical Quality Control Centers (Trial) issued in June 2009, indicators for the management and control of medical quality in tertiary general hospitals issued in 2011, and the Measures for the Management of Medical Quality formulated in 2016. These documents reflect the efforts and determination of China to improve the quality of care. To provide affordable and equitable access to high-quality health care for all Chinese citizens, the Chinese government launched a national health care reform to expand insurance coverage in 2009 [25]. China had basically achieved the goal of universal health coverage by the end of 2011, with the coverage rate reaching 95.7% [26,27]. Health insurance and the alleviation of cost-related barriers to health care have allowed tremendous progress in early diagnosis, high-quality treatment, and risk prevention [2]. Since 2009, an increasing number of hospitals in China have implemented “clinical pathway management,” with the goal of optimal sequencing and timing of interventions by physicians, nurses, and other staff for a particular diagnosis or procedure [28]. The policy most directly related to quality-of-care improvement was the “Tertiary General Hospital Accreditation Criteria” issued by the National Health Commission in 2011 [29]. More than 90% of the hospitals in our study were Tertiary General Hospitals. This tertiary status guaranteed the high-quality of hospital services and management.

A few indicators showed continuous and significant drops 2011-2017. Although the number of indicators that fell behind appears to be much lower compared with the number of indicators that achieved progress, underlying reasons still need discussion. Several potential factors may have contributed to the decline. First, there may still be remaining problems in the cooperation and communication between medical care providers and patients. Second, there were lags in case records that did not completely record the actual clinical operation. Third, hospitals differed in their understanding of the guidelines. Fourth, inconsistency among practitioners and recorders leads to inaccurate case records. Given the variation in care patterns across regions, further monitoring of QIs and establishment of incentive mechanisms are needed to ensure continued amelioration in care quality.

Of note, the utilization rate of antibacterial drugs increased, but the proportion of antibiotics selected in accordance with guidelines did not significantly improve, and instead worsened over time with a significant downward trend. Although China has focused on the management of antibacterial drugs [30], the phenomenon of improper use of antibacterial drugs still exists. Irrational use of antibacterial drugs for inpatients includes unreasonable selection of drugs, long course of medication, short interval of medication, and drug use without indication. Contributors to the irrational use of antibiotics are as follows. First, it is necessary to improve clinicians’ ability to use antibiotics reasonably. Second, the public’s awareness of rational use of antibiotics is low, and it is common to ask for antibiotics voluntarily in the course of medical treatment or in patients’ self-medication. It is necessary to standardize the process of drug use and strengthen the construction of a rational drug use monitoring system.

The improvement of care quality in China seems promising, but also faces challenges and obstacles. At the national level, documents and policies continuously emphasize the strengthening of care quality management, but the understanding of process quality management is insufficient at the hospital level, and the lagging informatisation of electronic medical records and statistical analyses meant timely feedback for the performance of QIs was unavailable in most hospitals. The available analysis and feedback in most hospitals mainly focused on efficiency indicators such as the bed turnover rate, in-hospital days, and cost. Therefore, establishing a qualified system for monthly or annual feedback on the performance of QIs would help hospitals and doctors to achieve targeted improvement. In addition, cooperation among different specialties within hospitals still needs enhancement, especially for cases requiring multidisciplinary diagnosis and treatment. Awareness among medical staff of quality improvement should be continuously strengthened to make the care quality the focus of hospitals’ routine management.

Several limitations should be noted when interpreting the present results. First, since the establishment of the monitoring system, there has been no specific intervention program for the performance of QIs. Therefore, although most indicators showed significant upward trends, many increases were only slight. Second, since the data were reported by hospitals voluntarily, new hospitals are constantly registered in the system, the composition of the hospital cohort in each year was different, and some newly-enrolled hospitals reported a relatively small number of records, selection bias would be difficult to ignore. We performed repeated sampling to ensure stability of trends by bootstrap; however, this might still have been unable to handle systematic bias in the hospital reporting process, and repeat sampling was not helpful in case of significant hospital selection bias, which required more accurate analysis over longer time-span. Third, due to the lack of patient-level covariates, this study was not adjusted for in the analysis. Although some studies have argued that analyses can be performed without risk adjustment for process indicators [31], we believe that the necessary risk adjustment is essential for accurate estimation, especially for outcome indicators, which might underestimate the EAPC of some hospitals. Fourth, due to missing data for some QIs, potential bias might exist in the present study to some extent. Fourth, it was difficult to track patients’ follow-up health information in the system; therefore, we could not determine the contribution of better adherence to QIs to long-term outcomes.

## CONCLUSIONS

In this analysis of National Specific (Single) Disease in China, we found the care quality in China has made major advances over time. However, the improvement in care in China is fairly unbalanced across geographic areas and this should be considered. The single disease monitoring system should constantly update evaluation indicators, increase the number of monitoring diseases, expand the monitoring coverage, and further monitor QIs. In addition, region-based approaches and incentive mechanisms are warranted to ensure continued advances in the quality of care in China.

## Additional material


Online Supplementary Document
