# Supplementary material for: Trends in care quality in China from 2011 to 2017: An analysis based on the National Specific (Single) Disease Monitoring System
Source: J Glob Health. 2023 Apr 28;13:04045. doi: 10.7189/jogh.13.04045 (PMC10141559; doi:10.7189/jogh.13.04045)
Supplement: Online Supplementary Document [file jogh-13-04045-s001.pdf]

Case reporting process

Data were extracted from the inpatient medical records, drug charts, discharge summary, and assessment sheets. Collectors could request additional materials such as laboratory reports stored on the computer, if they were missing from the clinical record. If the information remains unavailable, it was considered as real missing data. The system collected information on baseline demographic characteristics, diagnostic testing, detailed medication history, health care utilization, in-hospital outcomes, and satisfaction with hospital service quality. Collectors identified the target patients from medical records according to the International Classification of Diseases version 10, diagnosis codes. Eligible record IDs were loaded into a Microsoft Excel spreadsheet, randomized, and then selected consecutively. The proportion of cases selected for data reporting should not be less than 20%. The collectors were required to complete the data reporting at the end of each month.

Data reporting was performed by trained clinical data collectors using standardized definitions. Each participating hospital designated a surgeon or nurse to be responsible for (supervising) the data reporting on secure web forms. The collector needed to read the data reporting instructions carefully prior to performing the online data reporting. The system website provided a teaching process of data reporting with 5 cases and an assessment process with system-generated 10 cases. When the qualified rate achieved 95% or more, the collectors could start data reporting, otherwise a second assessment is required. To minimize error caused by manual reporting, the system automatically captured the information on the front-page of

inpatient medical records from the hospital information system via a Web-based data collection tool. Values that exceeded the expected ranges will be prompted for error notification. The system also provided predefined logic checks to identify errors or illogical data entries. Besides, annual feedback on data quality issues and quality-of-care would be provided to all registered hospitals.[1]

Study population

We extracted case records for the six diseases from the National Specific (Single) Disease Monitoring System from 2011 to 2017. As we aimed to assess trends in hospital quality improvement, we excluded drugs prescribed at discharge. Finally, 56 priority QIs were selected for the six studied diseases. These QIs were applied to eligible patients who had definite indications but no documented contraindications or other physician documentation of reasons was not supplied. Based on the completeness and accuracy of the case records, we excluded patients who were admitted via transfer and lacked documentation of outcomes at discharge. Patients with hospital admissions of less than 1 day were also excluded. To ensure the validity and stability of the HPCP, we also restricted the sample of hospitals: (1) ≥20 admissions; (2) eligible patients for all QIs selected for the present study; (3) ≥50 total care opportunities (the sum of the denominators of all QIs) and a non-zero-sum of correct care; and (4) facilities unable to provide hospital characteristics. Hospital region was classified as Western, Central, or Eastern according to China Census definitions.[2,3]

Denominator-based weighting method formula:

$$HPCP = \frac{n_1}{n_1 + n_2 + n_3 + \cdots + n_n} p_1 + \frac{n_2}{n_1 + n_2 + n_3 + \cdots + n_n} p_2 + \cdots + \frac{n_n}{n_1 + n_2 + n_3 + \cdots + n_n} p_n$$

$$= \frac{p_1 + p_2 + p_3 + \cdots + p_n}{n_1 + n_2 + n_3 + \cdots + n_n}$$

n: Indicator denominator (patients who are supposed to satisfy the requirement of that indicator)

p: Indicator numerator (target patients who meet the requirement of that indicator)

Table S1. Definition of quality indicators of acute myocardial infarction (AMI)

**Quality indicators**	**Numerators**	**Eligible definition (denominators)***
QI1: Use of aspirin or clopidogrel immediately on arrival	Patients with AMI who received aspirin after hospital arrival.	Patients with a diagnosis of AMI
QI2: PCI treatment within 90 minutes on arrival	Patients who received PCI treatment within 90 minutes on arrival	Patients with a diagnosis of STEMI or LBBB
QI3: Use of beta-blocker immediately on arrival	Patients who received β-blocker within 24 hours of disease onset	AMI patients without contraindications to β-blocker
QI4: Use of beta-blocker while in hospital	Patients who were prescribed beta-blocker while in hospital	AMI patients without contraindications to β-blocker
QI5: Use of aspirin/clopidogrel while in hospital	Patients who received aspirin/clopidogrel while in hospital	AMI patients without contraindications to aspirin/clopidogrel
QI6: Use of ACEI/ARB while in hospital	Patients who were prescribed ACEI/ARB while in hospital	AMI patients without contraindications to ACEI/ARB

QI7: Use of statins while in hospital.	Patients who were prescribed statins while in hospital	AMI patients without contraindications to statins
QI8: Continue to use beta-blocker after discharge	Patients who were prescribed beta-blocker after discharge	AMI patients without contraindications to β -blocker
QI9: Continue to use aspirin/clopidogrel after discharge	Patients who received aspirin/clopidogrel after discharge	AMI patients without contraindications to aspirin/clopidogrel
QI10: Continue to use ACEI/ARB after discharge	Patients who were prescribed ACEI/ARB after discharge	AMI patients without contraindications to ACEI/ARB
QI11: Continue to use statins after discharge	Patients who were prescribed statins after discharge	AMI patients without contraindications to statins
QI12: Smoking cessation, health counseling, and secondary prevention education	Patients who receive a health education	Patients with a diagnosis of AMI
QI13: In-hospital mortality	Patients who died during the hospitalization	Patients with a diagnosis of AMI.

* Eligible patients are those with definite indications but no documented contraindications (e.g.,

treatment intolerance, excessive risk of adverse reaction).

Abbreviation: AMI, Acute Myocardial Infarction; STEMI, ST-segment Elevation Myocardial Infarction; PCI, Percutaneous Transluminal Coronary Intervention; ACEI, Angiotesion Converting Enzyme Inhibitors; ARB, Angiotensin Receptor Blocker; LBBB, Left bundle branch block.

Table S2. Definition of quality indicators of coronary artery bypass grafting (CABG)

**Quality indicators**	**Numerators**	**Eligible definition (denominators)***
QI1: Use of the internal mammary artery (the first vessel graft)	Patients using the internal breast artery (internal thoracic artery) in the first vessel graft	Adult patients who underwent CABG
QI2: Reasonable selection of prophylactic antibiotics	Patients who used first-generation or second-generation cephalosporin in priority	Adult patients who underwent CABG
QI3: Use of preventive antibiotics within 1 hour before the operation	Patients who used the first dose of preventive antibiotics within 1 hour before the operation	Adult patients who underwent CABG
Q4: Stop using antibiotics within 72 hours after the operation	Patients without using antibiotics within 72 hours after the operation	Adult patients who underwent CABG
QI5: Health education	Patients who received health education	Adult patients who underwent CABG
QI6: In-hospital mortality	Patients who died during the hospitalization	Adult patients who underwent CABG

* Eligible patients are those with definite indications but no documented contraindications (e.g., treatment intolerance, excessive risk of adverse reaction).

Abbreviation: CABG, Coronary Artery Bypass Grafting.

Table S3. Definition of quality indicators of community-acquired pneumonia (CAP)

**Quality indicators**	**Numerators**	**Eligible definition (denominators)***
QI1: Etiology diagnosis for non-critical pneumonia	Patients whose blood collection were after admission or before the use of first antimicrobial	Patients with a diagnosis for non-critical pneumonia
QI2: Etiology diagnosis for critical pneumonia	Severe CAP patients whose CURB-65$\geqslant$3/ PSI$\geqslant$91 after admission or patients whose first blood collection were after admission to ICU	Patients diagnosed with severe CAP or with admission to ICU
QI3: Antibiotic treatment within 4 hours after admission to the hospital for non-critical pneumonia	Patients who received their first dose of antibiotics within 4 hours after hospital admission	Patients diagnosed with non-critical pneumonia
QI4: Antibiotic treatment within 4 hours after admission to the hospital for critical pneumonia	Severe CAP patients whose CURB-65$\geqslant$3/PSI$\geqslant$91 or patients who received their first dose of antibiotics within 4 hours after admission to ICU	Patients diagnosed with severe CAP or with admission to ICU
QI5: Selection of proper antibiotics for non-critical	Non-critical patients whose antimicrobial drug selection met	Patients diagnosed with non-critical pneumonia

patients conforms to the guidelines	the guidelines	
QI6: Selection of proper antibiotics for critical patients conforms to the guidelines	Critical patients whose antimicrobial drug selection met the guidelines	Patients diagnosed with severe CAP or with admission to ICU
QI7: Discharge within 14 days	Non-critical patients who met the discharge criteria and discharged criteria within 14 days	Patients diagnosed with non-critical pneumonia and discharged
QI8: In-hospital mortality	Patients who died during the hospitalization	Patients diagnosed with CAP

* Eligible patients are those with definite indications but no documented contraindications (e.g., treatment intolerance, excessive risk of adverse reaction).

Abbreviation: CAP: Community-acquired Pneumonia; ICU, intensive care unit; CURB, confusion, uremia, respiratory, rate blood pressure; PSI, Pneumonia severity Index.

Table S4. Definition of quality indicators of heart failure (HF)

**Quality indicators**	**Numerators**	**Eligible definition (denominators)***
QI1: Assessment of left ventricular function	Patients undergoing left ventricular function evaluation	Patients with a diagnosis of HF
QI2: Use of ACEI/ARB immediately on arrival	Patients using ACEI/ARB immediately on arrival	Patients with a diagnosis of HF
QI3: Use of beta-blocker as early as possible on arrival	Patients receiving their first dose of beta blockers within 24 hours of admission	Patients with beta-blocker indications
QI4: Use of diuretics and potassium while in hospital	Patients using diuretics and potassium while in hospital	Patients with a diagnosis of HF
QI5: Use of ACEI/ARB while in hospital	Patients using ACEI/ARB while in hospital	Patients with a diagnosis of HF
QI6: Use of beta-blocker while in hospital	Patients using beta-blocker while in hospital	Patients with a diagnosis of HF
QI7: Use of aldosterone antagonists while in hospital	Patients with severe heart failure using of aldosterone antagonists while in hospital	Patients with a diagnosis of HF
QI8: Continue to use diuretics and potassium after discharge	Patients continue to use diuretics and potassium after discharge	Patients with a diagnosis of HF
QI9: Continue to use	Patients continue to use	Patients with a diagnosis

ACEI/ARB after discharge	ACEI/ARB after discharge	of HF
QI10: Continue to use beta-blocker after discharge	Patients continue to use beta-blocker after discharge	Patients with a diagnosis of HF
QI11: Continue to use aldosterone antagonists after discharge	Patients continue to use aldosterone antagonists after discharge	Patients with a diagnosis of HF
QI12: Health education	Patients providing health education for patients	Patients with a diagnosis of HF
QI13: In-hospital mortality	Patients who died during the hospitalization	Patients with a diagnosis of HF

* Eligible patients are those with definite indications but no documented contraindications (e.g., treatment intolerance, excessive risk of adverse reaction).

Abbreviation: HF, Heart Failure; ACEI, Angiotensin converting enzyme inhibitors; ARB, angiotensin II receptor antagonists.

72 Table S5. Definition of quality indicators of Hip/Knee Replacement (H/K replacement)

Quality indicators	Numerators	Eligible definition (denominators)*
QI1: Selection of preventive antibiotics in line with specifications	Patients who used first-generation or second-generation cephalosporin in priority	Patients who underwent H/K replacement
QI2: Use of prophylactic antibiotic within 1 hour before the operation	Patients who used the first dose of prophylactic antibiotic within 1 hour before the operation	Patients who underwent H/K replacement
QI3: Use of additional antibiotics for operations that last for more than 3 hours	Patients who used of additional antibiotics	Patients whose operations last more than 3 hours or surgical blood transfusion more than 1500ml
QI4: Application of preventive prophylactic anticoagulants within 24 hours	Patients who were preventive prophylactic anticoagulants within 24 hours	Patients who underwent H/K replacement
QI5: Unilateral surgical blood transfusion more than 400ml/bilateral 800ml (reversed indicator)	Patients whose unilateral surgical blood transfusion more than 400ml/bilateral 800ml	Patients who underwent H/K replacement
QI6: Use of ACEI/ARB while	Patients who were prescribed	Patients who underwent

in hospital	ACEI/ARB while in hospital	H/K replacement
QI7: Discharge within 21 days.	Patients who discharge within 21 days	Patients who underwent H/K replacement

* Eligible patients are those with definite indications but no documented contraindications (e.g., treatment intolerance, excessive risk of adverse reaction).

Abbreviation: H/K Replacement, Hip/Knee Replacement; ACEI, Angiotesion Converting Enzyme Inhibitors; ARB, Angiotensin Receptor Blocker.

Table S6. Definition of quality indicators of acute ischemic stroke (AIS)

**Quality indicators**	**Numerators**	**Eligible definition (denominators)***
AIS-1. Thrombolytic therapy within 4.5 hours of symptom onset	Patients who have been provided with acute thrombolytic therapy within 4.5 hours of symptom onset	AIS patients who arrived within 2 hours after symptom onset
AIS-2. Anticoagulation in patients with atrial fibrillation	Patients who have been provided with anticoagulant therapy within 48 hours of admission	Patients with AIS and atrial fibrillation/flutter
AIS-3. Use of aspirin or clopidogrel within 48 hours after admission to hospital	Patients who have been provided with antiplatelet therapy within 48 hours of admission	Patients with a diagnosis of AIS.
AIS-4. Dysphagia assessment	Patients who have been provided with dysphagia screening before any oral intake during hospitalization.	Patients with a diagnosis of AIS.
AIS-5. Preventive of deep vein thrombosis (drug therapy)	Patients who have been provided with therapy to prevent venous thrombosis (medicine) within 48 hours of admission.	AIS patients who were not ambulating by hospital day two.
AIS-6. Preventive of deep vein	Patients who have been provided	AIS patients who were

thrombosis (physical therapy)	with therapy to prevent venous thrombosis (physics) within 48 hours of admission.	not ambulating by hospital day two.
AIS-7. Preventive of deep vein thrombosis (rehabilitation therapy)	Patients who have been provided with therapy to prevent venous thrombosis (rehabilitation therapy) within 48 hours of admission.	AIS patient who were not ambulating by hospital day two.
AIS-8. Health education	Patients who receive a health education	Patients with a diagnosis of AIS.
AIS-9. In-hospital mortality	Patients who died during the hospitalization	Patients with a diagnosis of AIS.

* Eligible patients are those with definite indications but no documented contraindications (e.g., treatment intolerance, excessive risk of adverse reaction).

Abbreviation: CT, Computed Tomography; MRI, Magnetic Resonance Imaging; AIS: Acute Ischemic Stroke; ECG, Electrocardiograph; rt-PA, recombinant tissue plasminogen activator.

Table S7. The characteristics of AMI's registered hospitals

Characteristic	1 year	2 years	3 years	4 years	5 years	6 years	7 years
**Ownership System, N (%)**							
Private	4(14.8)	2(7.4)	5(18.5)	2(7.4)	5(18.5)	4(14.8)	5(18.5)
Public	115(17.0)	105(15.5)	94(13.9)	73(10.8)	87(12.8)	73(10.8)	131(19.3)
**Category, N (%)**							
Specialty	6(13.3)	8(17.8)	9(20.0)	6(13.3)	6(13.3)	4(8.9)	6(13.3)
Comprehensive	114(17.2)	100(15.1)	90(13.6)	69(10.4)	86(13.0)	73(11.0)	130(19.6)
**Hospital level**							
Secondary	15(32.6)	17(37.0)	6(13.0)	1(2.2)	2(4.3)	2(4.3)	3(6.5)
Tertiary	105(15.9)	91(13.8)	93(14.1)	74(11.2)	90(13.6)	75(11.3)	133(20.1)
**Hospital grade, N (%)**							
Grade A	87(15.5)	83(14.7)	80(14.2)	63(11.2)	73(13.0)	66(11.7)	111(19.7)
Grade B	33(22.6)	26(17.8)	19(13.0)	12(8.2)	20(13.7)	11(7.5)	25(17.1)
**Affiliated with the Health and Construction Committee, N (%)**							
Not affiliated	119(17.2)	107(15.5)	94(13.6)	72(10.4)	91(13.2)	75(10.9)	132(19.1)
Affiliated	1(5.3)	2(10.5)	5(26.3)	3(15.8)	2(10.5)	2(10.5)	4(21.1)
**Affiliated University Hospital, N (%)**							
Not affiliated	91(17.4)	81(15.5)	70(13.4)	53(10.2)	71(13.6)	55(10.5)	101(19.3)
Affiliated	29(15.6)	27(14.5)	29(15.6)	22(11.8)	22(11.8)	22(11.8)	35(18.8)
**Subordination, N (%)**							
Municipal	70(17.0)	59(14.3)	52(12.6)	40(9.7)	53(12.9)	49(11.9)	89(21.6)
Provincial	27(14.1)	23(12.0)	31(16.2)	27(14.1)	28(14.7)	21(11.0)	34(17.8)
County	19(20.0)	26(27.4)	14(14.7)	6(6.3)	10(10.5)	7(7.4)	13(13.7)
**Actual Beds, median (IQR)**	1100.00(720.00-1500.00)	1151.00(826.00-1538.00)	1304.00(900.00-1800.00)	1371.00(881.00-1882.00)	1172.00(950.00-1688.00)	1492.50(1010.00-1944.00)	1462.00(1024.00-2023.00)
**ICU Beds, median (IQR)**	19.00(12.00-38.00)	24.00(12.00-36.00)	27.50(15.00-62.00)	25.50(16.00-58.00)	27.00(16.00-49.00)	33.50(16.00-69.00)	36.00(18.00-66.00)
**Special Needs Beds, median (IQR)**	0.00(0.00-23.00)	1.00(0.00-35.00)	4.50(0.00-29.00)	7.00(0.00-34.00)	0.00(0.00-14.00)	2.00(0.00-28.00)	8.00(0.00-31.00)
**Number of employees on duty, median (IQR)**	1408.00(1037.00-2106.00)	1595.00(1080.00-2061.00)	1799.00(1274.00-2610.00)	1769.50(1298.00-2667.00)	1668.50(1324.00-2360.00)	1994.00(1502.00-2558.00)	1999.00(1423.00-2742.00)
**Number of health technicians, median (IQR)**	1190.00(833.00-1709.00)	1350.00(953.00-1816.00)	1575.00(1123.00-2135.00)	1643.50(1092.00-2455.00)	1377.00(1093.00-2059.00)	1750.00(1254.00-2253.00)	1634.00(1210.00-2367.00)
**Number of physicians, median (IQR)**	398.50(292.00-624.00)	488.50(333.00-660.00)	530.00(373.00-752.00)	559.00(362.00-795.00)	462.00(362.00-655.00)	575.00(395.00-750.00)	543.00(412.00-785.00)
**Number of practicing physicians, median (IQR)**	396.50(291.00-621.50)	459.00(329.00-630.00)	523.00(367.00-740.00)	557.00(359.00-738.00)	456.00(352.00-655.00)	557.00(395.00-726.00)	541.50(404.50-783.00)

**Number of practicing Assistant Physicians, median (IQR)**	3.00(1.00-6.00)	3.00(0.00-6.00)	2.00(0.00-6.00)	1.00(0.00-4.00)	2.00(0.00-7.00)	3.00(1.00-9.50)	2.00(0.00-5.00)
**Number of nurses, median (IQR)**	629.00(436.00-877.00)	702.00(492.00-1004.00)	822.00(602.00-1188.00)	907.00(567.00-1344.00)	720.00(531.00-1124.00)	938.00(690.00-1224.00)	872.00(649.00-1283.00)
**Number of medical quality specialized staff, median (IQR)**	5.00(3.00-10.00)	8.00(3.00-14.00)	6.00(3.00-11.00)	6.00(4.00-11.00)	6.00(3.00-19.00)	5.00(3.00-11.00)	8.00(5.00-12.00)

Abbreviations: AMI, acute myocardial infarction; N, Number of hospitals; IQR, interquartile range.

Table S8. The characteristics of CABG's registered hospitals

Characteristic	1 year	2 years	3 years	4 years	5 years	6 years	7 years
**Ownership System, N (%)**							
Private	6(42.9)	2(14.3)	1(7.1)	2(14.3)	1(7.1)	2(14.3)	-
Public	75(25.7)	62(21.2)	42(14.4)	38(13.0)	21(7.2)	27(9.2)	27(9.2)
**Category, N (%)**							
Specialty	9(33.3)	3(11.1)	5(18.5)	5(18.5)	1(3.7)	-	4(14.8)
Comprehensive	72(25.8)	61(21.9)	38(13.6)	35(12.5)	21(7.5)	29(10.4)	23(8.2)
**Hospital level**							
Secondary	-	1(100.0)	-	-	-	-	-
Tertiary	81(26.6)	63(20.7)	43(14.1)	40(13.1)	22(7.2)	29(9.5)	27(8.9)
**Hospital grade, N (%)**							
Grade A	64(23.7)	57(21.1)	43(15.9)	37(13.7)	19(7.0)	26(9.6)	24(8.9)
Grade B	17(47.2)	7(19.4)	-	3(8.3)	3(8.3)	3(8.3)	3(8.3)
**Affiliated with the Health and Construction Committee, N (%)**							
Not affiliated	81(27.9)	62(21.4)	40(13.8)	35(12.1)	20(6.9)	27(9.3)	25(8.6)
Affiliated	-	2(12.5)	3(18.8)	5(31.3)	2(12.5)	2(12.5)	2(12.5)
**Affiliated University Hospital, N (%)**							
Not affiliated	58(29.1)	40(20.1)	23(11.6)	28(14.1)	12(6.0)	20(10.1)	18(9.0)
Affiliated	23(21.5)	24(22.4)	20(18.7)	12(11.2)	10(9.3)	9(8.4)	9(8.4)
**Subordination, N (%)**							
Municipal	49(28.0)	36(20.6)	20(11.4)	25(14.3)	12(6.9)	16(9.1)	17(9.7)
Provincial	25(21.7)	24(20.9)	22(19.1)	15(13.0)	8(7.0)	13(11.3)	8(7.0)
County	6(42.9)	3(21.4)	1(7.1)	-	2(14.3)	-	2(14.3)
**Actual Beds, median (IQR)**	1408.00(1059.00-1994.00)	1600.00(1004.00-2130.00)	1759.00(1500.00-2375.00)	1862.50(1149.00-2417.00)	1800.00(1304.00-2855.00)	2008.00(1290.00-3000.00)	1957.50(1580.00-2735.00)
**ICU Beds, median (IQR)**	31.00(20.00-58.00)	32.00(17.00-62.00)	53.00(29.00-90.50)	46.00(36.00-80.00)	42.00(25.50-97.50)	57.00(31.00-116.00)	80.50(34.00-112.50)
**Special Needs Beds, median (IQR)**	5.00(0.00-35.00)	15.50(0.00-50.00)	17.00(0.00-32.50)	14.00(0.00-42.00)	22.00(6.00-34.00)	12.00(0.00-72.00)	16.00(0.00-44.00)
**Number of employees on duty, median (IQR)**	1978.00(1534.00-2613.00)	2182.00(1639.00-2851.50)	2528.00(2296.00-3561.00)	2608.00(1715.00-3757.00)	2794.00(2178.50-3468.50)	2909.00(2150.00-3740.00)	2850.00(2336.00-4143.00)
**Number of health technicians, median (IQR)**	1723.50(1246.00-2308.00)	1819.00(1328.00-2323.00)	2173.00(1968.00-3114.00)	2383.00(1490.00-3183.00)	2264.00(1563.00-3272.00)	2450.00(1625.00-3445.00)	2493.00(1987.50-3609.50)
**Number of physicians, median (IQR)**	611.00(430.00-771.00)	625.50(432.00-779.50)	758.00(629.00-1018.00)	787.50(518.00-1011.00)	695.00(578.00-957.00)	847.00(538.00-1087.00)	782.50(577.50-1108.50)
**Number of practicing**	580.00(430.0	620.00(394.	737.00(628	782.50(517	719.00(576.	784.00(532.	778.00(571.5

**physicians, median (IQR)**	0-756.00)	00-740.00)	.00-1018.00)	.00-1010.50)	00-957.00)	00-1082.00)	0-1107.00)
**Number of practicing Assistant Physicians, median (IQR)**	1.00(0.00-5.00)	1.00(0.00-3.00)	0.00(0.00-1.00)	1.00(0.00-7.00)	2.00(0.00-5.00)	2.00(0.00-5.00)	0.00(0.00-3.00)
**Number of nurses, median (IQR)**	914.00(669.00-1218.00)	995.00(694.50-1232.50)	1216.50(1030.00-1722.00)	1165.00(774.00-1694.50)	1239.50(897.00-1556.00)	1126.00(961.00-1825.00)	1327.50(988.00-1904.00)
**Number of medical quality specialized staff, median (IQR)**	6.00(4.00-12.00)	8.50(4.00-15.00)	5.00(4.00-11.00)	5.00(3.00-16.00)	10.00(5.50-13.50)	9.00(7.00-16.50)	8.00(5.00-10.00)

Abbreviations: CABG, coronary artery bypass grafting; N, Number of hospitals; IQR, interquartile range.

Table S9. The characteristics of CAP's registered hospitals

Characteristic	1 year	2 years	3 years	4 years	5 years	6 years	7 years
**Ownership System, N (%)**							
Private	6(18.2)	6(18.2)	6(18.2)	6(18.2)	3(9.1)	4(12.1)	2(6.1)
Public	136(19.7)	115(16.6)	91(13.2)	70(10.1)	81(11.7)	93(13.5)	105(15.2)
**Category, N (%)**							
Specialty	9(20.9)	8(18.6)	9(20.9)	2(4.7)	5(11.6)	6(14.0)	4(9.3)
Comprehensive	134(19.6)	114(16.7)	89(13.0)	74(10.8)	79(11.5)	91(13.3)	103(15.1)
**Hospital level**							
Secondary	22(37.9)	19(32.8)	7(12.1)	2(3.4)	4(6.9)	2(3.4)	2(3.4)
Tertiary	119(17.9)	102(15.3)	91(13.7)	74(11.1)	80(12.0)	95(14.3)	105(15.8)
**Hospital grade, N (%)**							
Grade A	104(18.2)	86(15.1)	76(13.3)	63(11.1)	72(12.6)	77(13.5)	92(16.1)
Grade B	40(25.2)	36(22.6)	23(14.5)	13(8.2)	12(7.5)	20(12.6)	15(9.4)
**Affiliated with the Health and Construction Committee, N (%)**							
Not affiliated	142(19.9)	120(16.8)	96(13.5)	74(10.4)	82(11.5)	94(13.2)	105(14.7)
Affiliated	2(12.5)	2(12.5)	3(18.8)	2(12.5)	2(12.5)	3(18.8)	2(12.5)
**Affiliated University Hospital, N (%)**							
Not affiliated	108(19.9)	97(17.8)	73(13.4)	55(10.1)	60(11.0)	70(12.9)	81(14.9)
Affiliated	35(19.0)	25(13.6)	26(14.1)	21(11.4)	24(13.0)	27(14.7)	26(14.1)
**Subordination, N (%)**							
Municipal	77(18.2)	69(16.3)	48(11.3)	46(10.8)	47(11.1)	65(15.3)	72(17.0)
Provincial	35(19.3)	22(12.2)	27(14.9)	21(11.6)	24(13.3)	26(14.4)	26(14.4)
County	30(27.3)	27(24.5)	17(15.5)	9(8.2)	12(10.9)	6(5.5)	9(8.2)
**Actual Beds, median (IQR)**	1124.00(720.00-1574.00)	1010.00(750.00-1532.50)	1216.00(830.00-1688.00)	1200.00(962.00-1882.00)	1200.00(966.00-1768.00)	1369.00(1023.00-1880.00)	1575.50(1061.00-2023.00)
**ICU Beds, median (IQR)**	20.50(12.00-45.50)	19.50(12.00-40.50)	19.00(12.00-36.00)	27.50(17.50-49.50)	20.00(16.00-52.00)	31.00(18.00-65.00)	36.50(20.00-67.50)
**Special Needs Beds, median (IQR)**	2.00(0.00-20.00)	0.00(0.00-18.00)	10.00(0.00-35.00)	0.00(0.00-22.00)	0.00(0.00-24.00)	5.00(0.00-24.00)	3.00(0.00-31.00)
**Number of employees on duty, median (IQR)**	1448.00(918.00-2080.00)	1445.00(1077.00-2024.00)	1559.00(1147.50-2310.00)	1798.50(1347.00-2502.00)	1679.00(1253.00-2366.00)	1961.00(1483.00-2600.00)	2110.50(1496.50-2944.50)
**Number of health technicians, median (IQR)**	1213.00(760.00-1781.00)	1241.00(927.50-1799.00)	1268.50(977.00-1947.00)	1488.00(1099.00-2392.00)	1422.00(1105.00-2050.00)	1630.00(1210.00-2127.00)	1750.00(1283.00-2389.00)
**Number of physicians, median (IQR)**	398.00(285.00-653.00)	436.00(305.50-619.50)	458.50(352.50-671.50)	506.00(356.00-777.00)	460.00(335.00-676.00)	538.00(426.00-725.00)	588.00(419.00-826.00)
**Number of practicing physicians, median (IQR)**	396.00(278.00-653.00)	427.00(299.00-603.00)	420.00(326.00-661.00)	492.50(332.00-777.00)	456.00(330.00-676.00)	532.00(418.00-655.00)	579.00(419.00-796.00)

**Number of practicing Assistant Physicians, median (IQR)**	3.00(0.00-5.00)	3.00(0.00-6.50)	2.00(0.00-7.00)	2.00(0.00-6.50)	1.00(0.00-7.00)	3.00(0.00-6.00)	3.00(0.00-6.00)
**Number of nurses, median (IQR)**	659.50(441.00-975.00)	674.00(481.50-977.00)	712.00(508.00-1057.00)	783.00(584.00-1255.00)	720.00(559.00-1150.00)	861.00(666.00-1140.00)	923.00(692.00-1338.00)
**Number of medical quality specialized staff, median (IQR)**	6.00(3.00-10.00)	6.00(4.00-15.00)	4.00(3.00-9.00)	5.00(4.00-11.00)	5.50(4.00-10.50)	6.50(3.00-11.00)	8.00(5.00-15.50)

Abbreviations: CAP, community-acquired pneumonia; N, Number of hospitals; IQR, interquartile range.

Table S10. The characteristics of HF's registered hospitals

Characteristic	1 year	2 years	3 years	4 years	5 years	6 years	7 years
**Ownership System, N (%)**							
Private	7(24.1)	6(20.7)	1(3.4)	6(20.7)	7(24.1)	2(6.9)	-
Public	132(20.8)	121(19.0)	86(13.5)	69(10.8)	75(11.8)	63(9.9)	90(14.2)
**Category, N (%)**							
Specialty	8(19.0)	9(21.4)	10(23.8)	3(7.1)	2(4.8)	5(11.9)	5(11.9)
Comprehensive	132(21.1)	118(18.9)	78(12.5)	72(11.5)	80(12.8)	60(9.6)	85(13.6)
**Hospital level**							
Secondary	16(36.4)	15(34.1)	5(11.4)	1(2.3)	2(4.5)	2(4.5)	3(6.8)
Tertiary	124(19.9)	112(18.0)	83(13.3)	74(11.9)	80(12.8)	63(10.1)	87(14.0)
**Hospital grade, N (%)**							
Grade A	111(20.9)	96(18.0)	72(13.5)	59(11.1)	67(12.6)	54(10.2)	73(13.7)
Grade B	29(21.3)	31(22.8)	16(11.8)	17(12.5)	15(11.0)	11(8.1)	17(12.5)
**Affiliated with the Health and Construction Committee, N (%)**							
Not affiliated	138(21.2)	125(19.2)	83(12.8)	73(11.2)	80(12.3)	64(9.8)	87(13.4)
Affiliated	2(11.1)	2(11.1)	5(27.8)	3(16.7)	2(11.1)	1(5.6)	3(16.7)
**Affiliated University Hospital, N (%)**							
Not affiliated	106(21.5)	91(18.4)	64(13.0)	53(10.7)	65(13.2)	49(9.9)	66(13.4)
Affiliated	34(19.5)	36(20.7)	24(13.8)	23(13.2)	17(9.8)	16(9.2)	24(13.8)
**Subordination, N (%)**							
Municipal	80(20.9)	64(16.7)	45(11.7)	44(11.5)	49(12.8)	45(11.7)	56(14.6)
Provincial	31(17.4)	36(20.2)	27(15.2)	25(14.0)	22(12.4)	14(7.9)	23(12.9)
County	25(26.3)	24(25.3)	14(14.7)	4(4.2)	11(11.6)	6(6.3)	11(11.6)
**Actual Beds, median (IQR)**	1050.00(791.00-1475.00)	1195.00(818.00-1710.00)	1374.00(1000.00-1863.00)	1200.00(801.00-1695.00)	1280.50(1000.00-1655.00)	1401.00(1039.00-2174.00)	1488.00(1000.00-2023.00)
**ICU Beds, median (IQR)**	22.50(12.00-41.00)	26.00(13.00-53.00)	20.00(15.00-43.00)	24.00(13.00-52.00)	26.00(16.00-64.00)	36.00(20.00-72.50)	36.00(17.00-62.00)
**Special Needs Beds, median (IQR)**	1.00(0.00-20.00)	5.00(0.00-30.00)	0.00(0.00-22.00)	0.00(0.00-16.00)	0.00(0.00-20.00)	2.00(0.00-30.50)	18.00(0.00-35.00)
**Number of employees on duty, median (IQR)**	1468.00(1016.00-2080.00)	1640.00(1123.00-2290.00)	1763.00(1338.00-2608.00)	1647.00(1188.00-2395.50)	1794.00(1367.00-2301.00)	2041.00(1502.00-2911.00)	2029.00(1414.00-2999.00)
**Number of health technicians, median (IQR)**	1223.50(878.00-1800.00)	1434.50(988.00-1932.50)	1490.00(1099.00-2289.00)	1413.00(972.00-2059.00)	1562.00(1201.00-2005.00)	1707.00(1254.00-2469.00)	1656.50(1216.50-2394.50)
**Number of physicians, median (IQR)**	437.00(325.00-653.00)	497.00(357.00-708.00)	527.00(365.00-779.00)	462.00(338.50-653.50)	534.50(389.00-694.00)	584.00(423.50-806.00)	551.00(392.00-796.00)
**Number of practicing physicians, median (IQR)**	425.00(318.00-637.00)	495.50(341.00-708.00)	503.00(363.00-766.00)	454.50(329.00-633.50)	523.00(387.00-691.00)	593.00(415.00-796.00)	546.00(386.00-796.00)

**Number of practicing Assistant Physicians, median (IQR)**	3.00(1.00-7.00)	2.00(0.00-7.00)	2.00(0.00-5.00)	2.00(0.00-7.00)	3.00(0.00-7.00)	2.00(0.00-11.00)	2.00(0.00-5.00)
**Number of nurses, median (IQR)**	660.00(487.00-919.00)	767.50(525.00-1083.00)	826.00(593.00-1271.00)	736.00(520.50-1116.50)	829.00(590.00-1115.00)	905.00(666.00-1312.50)	872.00(620.00-1338.00)
**Number of medical quality specialized staff, median (IQR)**	5.00(3.00-10.00)	7.00(3.00-15.00)	5.50(4.00-9.00)	8.00(4.00-19.00)	5.00(3.00-16.00)	8.00(4.00-13.00)	7.00(4.00-12.00)

Abbreviations: HF, heart failure; N, Number of hospitals; IQR, interquartile range.

Table S11. The characteristics of H/K's registered hospitals

Characteristic	1 year	2 years	3 years	4 years	5 years	6 years	7 years
**Ownership System, N (%)**							
Private	6(21.4)	4(14.3)	5(17.9)	4(14.3)	3(10.7)	3(10.7)	3(10.7)
Public	112(17.2)	105(16.1)	82(12.6)	68(10.4)	79(12.1)	99(15.2)	107(16.4)
**Category, N (%)**							
Specialty	6(17.1)	8(22.9)	4(11.4)	6(17.1)	4(11.4)	2(5.7)	5(14.3)
Comprehensive	113(17.4)	102(15.7)	83(12.8)	67(10.3)	78(12.0)	100(15.4)	105(16.2)
**Hospital level**							
Secondary	12(34.3)	9(25.7)	4(11.4)	4(11.4)	2(5.7)	2(5.7)	2(5.7)
Tertiary	105(16.3)	101(15.6)	83(12.8)	69(10.7)	80(12.4)	100(15.5)	108(16.7)
**Hospital grade, N (%)**							
Grade A	90(16.5)	82(15.1)	67(12.3)	63(11.6)	61(11.2)	85(15.6)	96(17.6)
Grade B	29(20.7)	28(20.0)	20(14.3)	11(7.9)	21(15.0)	17(12.1)	14(10.0)
**Affiliated with the Health and Construction Committee, N (%)**							
Not affiliated	118(17.7)	106(15.9)	85(12.8)	73(11.0)	79(11.9)	102(15.3)	103(15.5)
Affiliated	1(5.6)	4(22.2)	2(11.1)	1(5.6)	3(16.7)	-	7(38.9)
**Affiliated University Hospital, N (%)**							
Not affiliated	96(19.0)	81(16.1)	61(12.1)	54(10.7)	62(12.3)	75(14.9)	75(14.9)
Affiliated	23(12.8)	29(16.1)	26(14.4)	20(11.1)	20(11.1)	27(15.0)	35(19.4)
**Subordination, N (%)**							
Municipal	69(17.1)	58(14.4)	48(11.9)	42(10.4)	45(11.2)	65(16.1)	76(18.9)
Provincial	29(16.1)	34(18.9)	22(12.2)	16(8.9)	26(14.4)	25(13.9)	28(15.6)
County	17(19.3)	17(19.3)	14(15.9)	11(12.5)	11(12.5)	12(13.6)	6(6.8)
**Actual Beds, median (IQR)**	1112.00(742.00-1580.00)	1213.00(855.00-1901.00)	1200.00(900.00-1544.00)	1210.00(968.00-1677.00)	1275.00(1053.00-1750.50)	1385.00(1000.00-1800.00)	1688.50(1099.00-2200.00)
**ICU Beds, median (IQR)**	16.00(11.00-36.00)	23.50(12.50-51.00)	21.00(15.00-40.00)	30.00(17.00-58.00)	29.00(13.00-53.00)	30.00(18.00-58.00)	39.00(22.00-78.00)
**Special Needs Beds, median (IQR)**	4.00(0.00-34.00)	1.00(0.00-20.00)	0.00(0.00-18.00)	0.00(0.00-22.00)	0.00(0.00-22.00)	2.00(0.00-36.00)	12.50(0.00-34.50)
**Number of employees on duty, median (IQR)**	1446.50(1106.00-2031.00)	1694.00(1199.50-2674.00)	1517.50(1249.50-1992.00)	1772.00(1260.00-2290.00)	1857.00(1325.00-2417.00)	1920.50(1486.50-2392.00)	2227.00(1583.00-3214.00)
**Number of health technicians, median (IQR)**	1228.00(936.00-1781.00)	1405.00(973.00-2324.00)	1284.00(1115.00-1709.00)	1530.00(1037.00-1870.00)	1554.00(1052.00-2132.00)	1626.50(1242.50-2032.50)	1987.00(1421.00-2935.00)
**Number of physicians, median (IQR)**	415.00(314.00-637.00)	538.00(333.00-755.00)	449.50(368.50-609.00)	521.00(356.00-697.00)	495.00(329.00-707.00)	539.00(420.00-657.00)	661.00(482.00-957.00)
**Number of practicing physicians, median (IQR)**	405.00(308.00-630.00)	504.00(334.00-740.00)	445.50(367.50-579.00)	506.00(324.00-697.00)	488.50(327.00-661.00)	524.00(409.50-649.00)	655.00(473.00-957.00)

**Number of practicing Assistant Physicians, median (IQR)**	3.00(0.00-6.00)	1.50(0.00-4.00)	2.00(0.00-6.00)	2.00(0.00-9.00)	2.00(0.00-7.00)	3.00(1.00-7.00)	2.00(0.00-5.00)
**Number of nurses, median (IQR)**	666.50(436.00-979.00)	753.00(572.00-1260.00)	693.00(562.50-902.00)	802.00(550.00-1068.00)	749.00(528.00-1123.00)	872.00(635.00-1121.00)	1053.00(744.00-1527.00)
**Number of medical quality specialized staff, median (IQR)**	5.00(3.00-12.00)	6.00(3.00-11.00)	5.00(3.00-10.00)	5.00(3.50-11.00)	7.00(4.00-15.00)	7.50(4.00-11.00)	8.00(5.00-13.00)

Abbreviations: H/K, hip/knee replacement; N, Number of hospitals; IQR, interquartile range.

Table S12. The characteristics of AIS's registered hospitals

Characteristic	1 year	2 years	3 years	4 years	5 years	6 years	7 years
**Ownership System, N (%)**							
Private	3(12.5)	6(25.0)	3(12.5)	1(4.2)	5(20.8)	2(8.3)	4(16.7)
Public	71(13.9)	74(14.5)	54(10.5)	57(11.1)	62(12.1)	74(14.5)	120(23.4)
**Category, N (%)**							
Specialty	2(7.1)	5(17.9)	6(21.4)	3(10.7)	3(10.7)	7(25.0)	2(7.1)
Comprehensive	73(14.3)	75(14.7)	52(10.2)	55(10.8)	64(12.5)	69(13.5)	122(23.9)
**Hospital level**							
Secondary	10(27.8)	9(25.0)	4(11.1)	2(5.6)	6(16.7)	2(5.6)	3(8.3)
Tertiary	63(12.6)	71(14.2)	54(10.8)	55(11.0)	61(12.2)	74(14.8)	121(24.2)
**Hospital grade, N (%)**							
Grade A	60(14.0)	60(14.0)	43(10.0)	48(11.2)	56(13.1)	60(14.0)	101(23.6)
Grade B	15(13.4)	20(17.9)	16(14.3)	10(8.9)	11(9.8)	17(15.2)	23(20.5)
**Affiliated with the Health and Construction Committee, N (%)**							
Not affiliated	75(14.2)	79(14.9)	58(11.0)	55(10.4)	65(12.3)	77(14.6)	120(22.7)
Affiliated	-	1(9.1)	1(9.1)	3(27.3)	2(18.2)	-	4(36.4)
**Affiliated University Hospital, N (%)**							
Not affiliated	60(15.3)	58(14.8)	42(10.7)	39(9.9)	45(11.5)	57(14.5)	91(23.2)
Affiliated	15(10.2)	22(15.0)	16(10.9)	19(12.9)	22(15.0)	20(13.6)	33(22.4)
**Subordination, N (%)**							
Municipal	47(14.2)	52(15.8)	32(9.7)	33(10.0)	36(10.9)	49(14.8)	81(24.5)
Provincial	16(12.2)	18(13.7)	16(12.2)	12(9.2)	20(15.3)	19(14.5)	30(22.9)
County	12(17.4)	9(13.0)	7(10.1)	11(15.9)	9(13.0)	8(11.6)	13(18.8)
**Actual Beds, median (IQR)**	1200.00(800.00-1695.00)	1200.00(718.00-1709.50)	1100.00(765.00-1499.00)	1497.50(990.00-1997.00)	1165.00(828.50-1765.00)	1325.00(1172.00-1800.00)	1304.00(900.00-1830.00)
**ICU Beds, median (IQR)**	22.00(16.00-52.00)	23.00(14.00-53.00)	14.00(10.00-37.00)	38.00(20.00-67.50)	22.00(14.00-45.00)	24.50(16.00-58.50)	31.00(14.00-54.00)
**Special Needs Beds, median (IQR)**	3.50(0.00-26.00)	2.00(0.00-30.00)	0.00(0.00-20.00)	0.00(0.00-19.50)	0.00(0.00-1.00)	10.50(0.00-35.00)	0.50(0.00-35.00)
**Number of employees on duty, median (IQR)**	1501.00(1102.00-2339.00)	1653.00(1009.50-2485.50)	1421.00(984.00-2024.00)	1986.00(1387.00-2677.50)	1521.00(1194.00-2247.50)	1863.50(1557.00-2407.00)	1862.50(1353.00-2603.00)
**Number of health technicians, median (IQR)**	1326.00(896.00-2059.00)	1276.50(893.00-2172.50)	1229.00(929.00-1729.00)	1741.00(1189.00-2427.00)	1274.00(957.00-1873.50)	1569.00(1238.00-2059.00)	1599.00(1220.00-2289.00)
**Number of physicians, median (IQR)**	417.00(287.00-681.00)	453.00(289.00-798.00)	452.00(307.00-619.00)	649.00(398.00-825.50)	460.00(340.00-595.00)	562.00(428.50-762.50)	527.50(396.00-708.00)
**Number of practicing physicians, median (IQR)**	409.00(268.00-677.00)	449.00(289.00-728.00)	429.00(306.00-561.00)	606.00(395.00-807.00)	455.00(329.00-593.00)	544.00(420.00-769.00)	527.50(385.00-704.00)

**Number of practicing Assistant Physicians, median (IQR)**	2.00(0.00-5.00)	3.00(0.00-4.00)	3.00(0.00-7.00)	1.00(0.00-11.00)	2.00(0.00-7.00)	3.00(0.00-9.00)	3.00(0.00-5.50)
**Number of nurses, median (IQR)**	721.50(452.00-1103.00)	657.00(451.00-1157.00)	740.00(452.00-975.00)	932.50(646.00-1347.50)	702.00(491.00-1023.00)	822.50(679.50-1105.00)	869.50(620.00-1205.00)
**Number of medical quality specialized staff, median (IQR)**	6.00(3.00-15.00)	6.00(3.00-15.00)	6.00(3.50-10.00)	7.00(4.00-10.00)	5.50(4.00-10.00)	7.00(3.00-13.00)	6.00(4.00-12.00)

Abbreviations: AIS, acute ischemic stroke; N, Number of hospitals; IQR, interquartile range.

Table S13. The QIs utilization rate of AMI in 2011 and 2017, and percentage changes in absolute number and EAPCs at both nation and region

Disease	QI	Rate in 2011	Rate in 2017	Numerators	National		EAST		Central		West	
					EAPC	95%*CI*	EAPC	95%*CI*	EAPC	95%*CI*	EAPC	95%*CI*
AMI	QI1	52.32	59.89	29424	1.54	-1.48-4.64	0.59	-1.48-2.71	3.94	-1.68-9.87	2.72	-1.69-7.33
	QI2	57.96	69.91	18720	4.21	1.37-7.13	3.22	0.1-6.43	4.64	-1.09-10.71	8.71	6.47-11.01
	QI3	83.28	54.65	72183	-9.00	-16.37--0.97	-8.20	-15.63--0.12	-11.22	-19.36--2.25	-10.09	-18.29--1.07
	QI4	99.03	99.11	114151	0.03	-0.05-0.11	0.03	-0.07-0.12	0.11	-0.16-0.37	-0.03	-0.2-0.14
	QI5	88.51	93.87	82539	1.23	0.60-1.87	1.49	0.7-2.28	0.45	-0.45-1.37	0.89	0.18-1.62
	QI6	86.68	83.7	83934	-0.71	-1.62-0.22	-0.61	-1.45-0.24	-1.3	-2.33--0.25	-0.75	-2.16-0.69
	QI7	96.78	98.69	103994	0.40	0.21-0.58	0.42	0.22-0.62	0.38	0.03-0.74	0.29	0.08-0.51
	QI8	95.8	96.87	111484	0.13	-0.12-0.38	0.23	-0.06-0.51	-0.27	-0.51--0.02	0.16	-0.1-0.42
	QI9	86.77	92.91	81923	1.24	0.70-1.79	1.53	0.84-2.22	0.2	-0.39-0.8	1.09	0.26-1.92
	QI10	84.26	81.11	82780	-0.78	-1.65-0.10	-0.75	-1.54-0.04	-1.74	-2.84--0.64	-0.23	-1.64-1.2
	QI11	94.92	96.67	112488	0.28	0.01-0.55	0.43	0.13-0.73	-0.22	-0.57-0.14	0.16	-0.06-0.39
	QI12	98.48	99.39	107411	0.18	-0.09-0.46	0.16	0.05-0.27	0.46	-1.39-2.35	0.02	-0.06-0.11
	QI13	4.72	2.02	3636	-14.27	-17.20--11.24	-17.73	-20.96--14.37	-8.42	-19.54-4.23	-11.44	-17.4--5.05
	HPCP	90.03	87.36	1004667	-0.89	-2.00-0.23	-0.78	-1.97-0.42	-1.34	-2.29--0.37	-0.98	-2.39-0.45

Abbreviations: AMI, acute myocardial infarction; QI, quality indicator; HPCP, hospital process composite performance; EAPC, estimated annual percentage change; CI, confidence interval.

Table S14. The QIs utilization rate of CABG in 2011 and 2017, and percentage changes in absolute number and EAPCs at both nation and region

**Disease**	**QI**	**Rate in 2011**	**Rate in 2017**	**Numerators**	**National**		**EAST**		**Central**		**West**	
					**EAPC**	**95%*CI***	**EAPC**	**95%*CI***	**EAPC**	**95%*CI***	**EAPC**	**95%*CI***
CABG	QI1	0.75	0.79	20413	0.74	-0.27-1.75	0.11	-1.53-1.78	0.25	-1.71-2.26	9.5	2.46-17.03
	QI2	0.71	0.91	33040	3.03	-0.51-6.70	2.71	-1.75-7.37	3.86	2.35-5.38	2.52	0.37-4.72
	QI3	0.77	0.82	15623	1.23	-2.28-4.87	1.20	-3.82-6.49	-3.03	-8.63-2.91	3.55	0.04-7.18
	QI4	0.20	0.53	10637	11.97	-0.46-25.96	6.68	-7.63-23.2	48.36	15.92-89.87	-0.59	-23.26-28.77
	QI5	0.78	0.94	37052	0.90	-4.90-7.05	1.96	-3.33-7.54	-3.64	-15.45-9.83	1.78	-5.14-9.21
	QI6	1.80	0.93	416	-16.91	-28.28--3.74	-20.80	-34.4--4.40	-6.84	-20.35-8.96	-22.2	-44.37-8.79
	HPCP	0.73	0.86	117181	2.23	0.83-3.64	1.87	-1.97-0.42	2.36	-0.08-4.86	3.36	0.86-5.92

Abbreviations: CABG, coronary artery bypass graft; QI, quality indicator; HPCP, hospital process composite performance; EAPC, estimated annual percentage change; CI, confidence interval.

Table S15. The QIs utilization rate of CAP in 2011 and 2017, and percentage changes in absolute number and EAPCs at both nation and region

Disease	QI	Rate in 2011	Rate in 2017	Numerators	National		EAST		Central		West	
					**EAPC**	**95%*CI***	**EAPC**	**95%*CI***	**EAPC**	**95%*CI***	**EAPC**	**95%*CI***
CAP	QI1	48.08	87.56	94526	12.45	7.76-17.35	11.58	6.87-16.49	13.99	9.48-18.68	13.51	8.51-18.73
	QI2	65.62	95.32	10207	7.75	5.24-10.33	8.22	4.36-12.23	4.35	1.04-7.77	8.10	4.49-11.84
	QI3	68.1	92.26	128435	6.55	3.39-9.81	5.60	2.07-9.25	13.24	8.18-18.54	4.99	1.57-8.52
	QI4	88.13	89.86	10490	0.79	-0.08-1.66	1.17	-0.72-3.09	0.88	-2.24-4.10	-0.68	-2.82-1.51
	QI5	89.21	91.04	12281	0.61	0.00-1.22	1.92	0.42-3.44	-1.43	-3.87-1.07	-3.82	-6.43--1.14
	QI6	92.05	62.39	124820	-8.75	-14.04--3.14	-8.17	-15.7-0.03	-13.61	-18.56--8.36	-7.43	-10.38--4.39
	QI7	77.67	87.46	207604	1.92	1.29-2.55	1.80	1.05-2.57	1.78	1.09-2.47	2.62	1.26-3.99
	QI8	1.44	0.39	1605	-19.57	-24.22--14.63	-16.68	-22.62--10.30	-21.49	-29.81--12.19	-24.94	-35.19--13.06
	HPCP	78.33	86.42	589968	1.69	1.44-1.95	1.56	0.71-2.41	1.88	1.49-2.28	1.94	1.03-2.85

Abbreviations: CAP, community-acquired pneumonia; QI, quality indicator; HPCP, hospital process composite performance; EAPC, estimated annual percentage change; CI, confidence interval.

Table S16. The QIs utilization rate of HF in 2011 and 2017, and percentage changes in absolute number and EAPCs at both nation and region

Disease	QI	Rate in 2011	Rate in 2017	Numerators	National		EAST		Central		West	
					EAPC	95%*CI*	EAPC	95%*CI*	EAPC	95%*CI*	EAPC	95%*CI*
HF	QI1	52.4	70.98	103252	5.68	4.19-7.18	6.75	3.43-10.17	3.35	-0.39-7.23	4.61	0.30-9.11
	QI2	86.74	80.15	40520	-1.04	-2.23-0.17	-0.62	-2.07-0.87	0.48	-0.24-1.21	-2.89	-5.35--0.36
	QI3	72.92	77.27	52287	1.21	0.16-2.27	1.51	0.26-2.77	1.89	-1.20-5.07	0.01	-2.73-2.82
	QI4	93.1	92.51	113980	0.10	-0.54-0.74	0.06	-0.80-0.92	0.26	-0.33-0.84	0.15	-1.22-1.54
	QI5	55.99	72.72	92303	4.07	-0.96-9.36	3.64	-1.14-8.65	3.81	-0.99-8.84	5.50	-0.87-12.27
	QI6	63.38	68.94	77203	1.39	0.95-1.82	1.84	1.21-2.48	1.59	-0.70-3.93	0.71	-1.00-2.46
	QI7	83.38	86.92	102205	0.80	-0.39-2.01	0.41	-0.98-1.82	0.22	-0.72-1.17	2.23	0.30-4.19
	QI8	87.49	87.14	107907	0.24	-0.85-1.35	0.15	-1.43-1.76	-0.02	-0.72-0.68	0.78	-1.19-2.78
	QI9	78.56	71.05	96862	-1.96	-2.59--1.33	-2.01	-2.44--1.59	-2.47	-4.97-0.10	-1.10	-2.66-0.50
	QI10	64.56	68.82	77797	1.11	0.50-1.72	1.69	0.79-2.59	0.51	-1.72-2.80	0.77	-0.91-2.48
	QI11	79.71	82.53	98147	0.75	-0.76-2.27	0.31	-1.71-2.37	0.25	-0.63-1.13	2.45	0.23-4.72
	QI12	68.01	56.74	107925	-3.34	-5.57--1.07	-2.92	-5.76-0.01	-4.75	-13.61-5.01	-3.50	-6.55--0.34
	QI13	1.38	0.82	1504	-8.43	-16.46-0.38	-8.86	-17.53-0.73	-2.11	-12.35-9.32	-11.58	-22.94-1.44
	HPCP	74.7	77.39	1071892	0.60	-0.42-1.62	0.66	-0.49-1.82	0.21	-1.86-2.32	0.80	-0.46-2.08

Abbreviations: HF, heart failure; QI, quality indicator; HPCP, hospital process composite performance; EAPC, estimated annual percentage change; CI, confidence interval.

Table S17. The QIs utilization rate of H/K in 2011 and 2017, and percentage changes in absolute number and EAPCs at both nation and region

**Disease**	**QI**	**Rate in 2011**	**Rate in 2017**	**Numerators**	**National**		**EAST**		**Central**		**West**	
					**EAPC**	**95%*CI***	**EAPC**	**95%*CI***	**EAPC**	**95%*CI***	**EAPC**	**95%*CI***
H/K	QI1	82.17	83.74	125165	0.55	-1.34-2.48	-0.02	-2.28-2.30	2.02	-0.27-3.81	1.56	-0.32-3.48
	QI2	73.33	72.83	67322	-0.97	-3.25-1.35	-3.15	-5.80—0.44	5.96	1.08-11.07	1.65	0.04-3.29
	QI3	48.54	60.09	2998	2.15	-3.12-7.70	1.31	-3.83-6.73	1.27	-12.71-17.49	3.90	-3.54-11.90
	QI4	69.91	78.36	125333	1.87	0.16-3.60	3.01	0.11-5.99	5.35	2.86-7.91	-3.46	-11.32-5.10
	QI5	41.85	19.28	19483	-12.60	-15.53--9.57	18.08	-20.20--15.90	-7.50	-11.72--3.08	-5.65	-20.46-11.91
	QI6	68.87	75.62	118017	1.68	0.31-3.06	2.41	0.32-4.54	2.45	-3.01-8.21	-0.71	-2.93-1.56
	QI7	74.94	84.37	80796	6.99	-3.29-18.36	8.98	-6.61-27.17	6.48	-6.82-21.69	7.17	-6.09-22.32
	HPCP	71.2	79.68	539114	2.04	1.49-2.61	2.20	0.95-3.46	4.55	2.52-6.61	0.85	-0.56-2.28

Abbreviations: H/K, hip/knee replacement; QI, quality indicator; HPCP, hospital process composite performance; EAPC, estimated annual percentage change; CI, confidence interval.

Table S18. The QIs utilization rate of AIS in 2011 and 2017, and percentage changes in absolute number and EAPCs at both nation and region

**Disease**	**QI**	**Rate in 2011**	**Rate in 2017**	**Numerators**	**National**		**EAST**		**Central**		**West**	
					**EAPC**	**95%*CI***	**EAPC**	**95%*CI***	**EAPC**	**95%*CI***	**EAPC**	**95%*CI***
AIS	QI1	0.43	0.59	1246	4.07	-0.53-8.88	6.01	0.05-12.32	4.95	-8.48-20.35	-13.44	-24.98--0.11
	QI2	0.34	0.39	16466	-0.63	-7.82-7.11	-1.91	-9.33-6.12	13.22	5.19-21.86	-9.02	-19.51-2.85
	QI3	0.98	0.99	289559	0.06	-0.03-0.16	0.04	-0.03--0.11	-0.03	-0.18-0.11	0.27	0.08-0.46
	QI4	0.30	0.53	194800	9.78	8.88-10.68	9.76	7.09-12.50	16.31	9.82-23.19	6.44	3.11-9.88
	QI5	0.25	0.33	17504	5.90	3.42-8.43	7.08	4.66-9.56	0.96	-9.26-12.33	10.75	1.42-20.93
	QI6	0.26	0.49	16043	11.77	7.44-16.28	12.77	8.93-16.75	6.06	-2.76-15.69	13.93	-2.93-33.72
	QI7	0.84	0.85	58361	0.21	-0.82-1.25	0.45	-2.04-3.01	1.17	-1.33-3.74	-0.34	-2.99-2.38
	QI8	0.40	0.54	211468	3.78	0.91-6.73	4.37	0.13-8.80	7.93	-1.06-17.74	0.06	-1.76-1.92
	QI9	1.54	0.44	3022	-19.70	-25.79--13.11	-24.13	-27.82--20.25	-13.31	-27.65-3.86	-19.62	-28.30--9.89
	HPCP	0.58	0.74	808469	3.91	3.20-4.62	3.98	2.31-5.67	6.43	3.50-9.45	2.19	1.63-2.75

Abbreviations: AIS, acute ischemic stroke; QI, quality indicator; HPCP, hospital process composite performance; EAPC, estimated annual percentage change; CI, confidence interval.

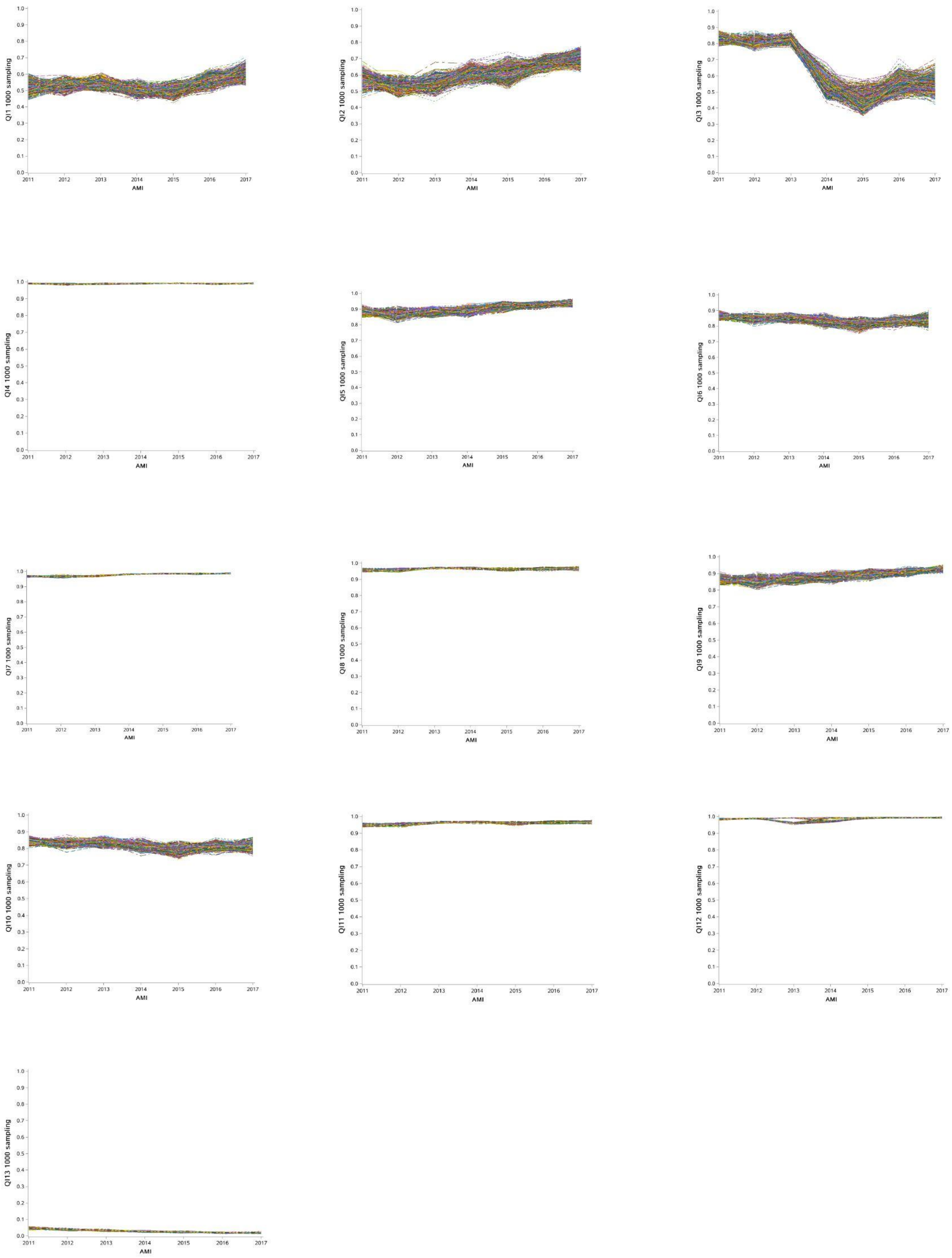

Figure S1. Trends in QIs utilization rate of AMI from 2011 to 2017 by bootstrap method

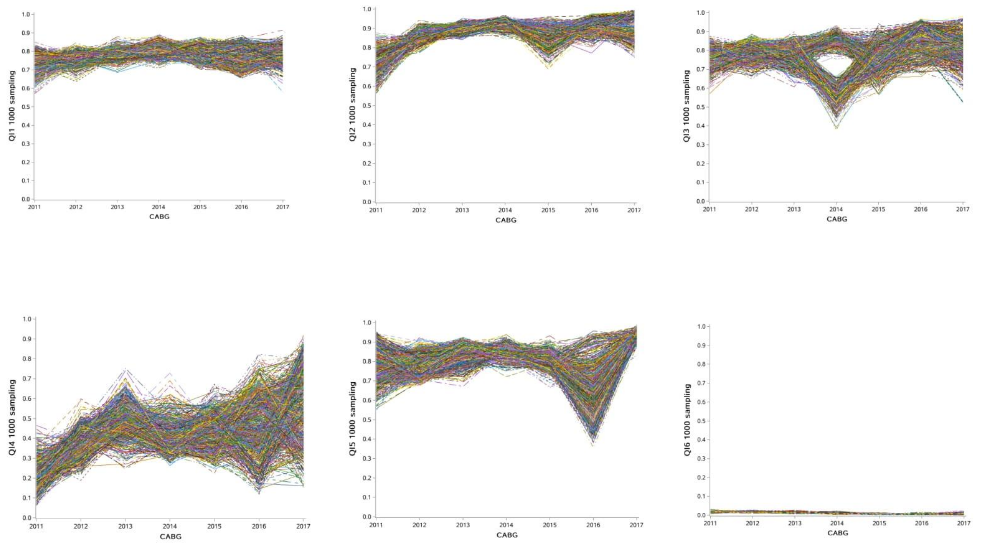

Figure S2. Trends in QIs utilization rate of CABG from 2011 to 2017 by bootstrap method

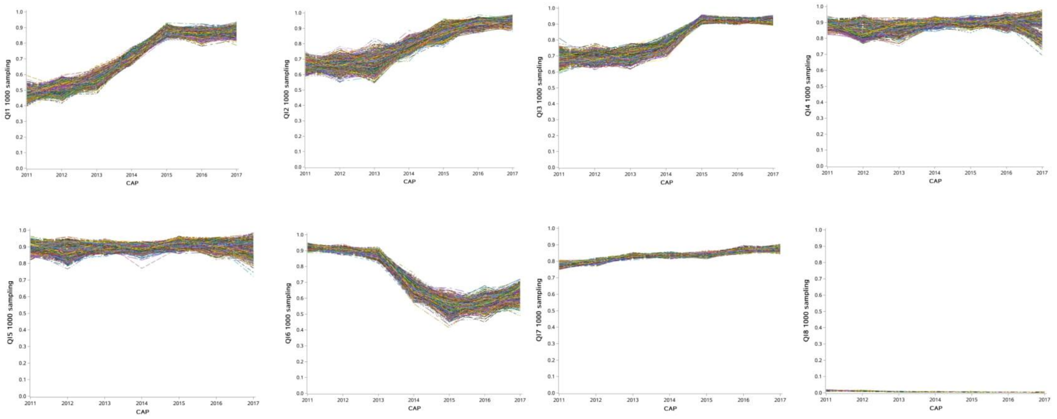

Figure S3. Trends in QIs utilization rate of CAP from 2011 to 2017 by bootstrap method

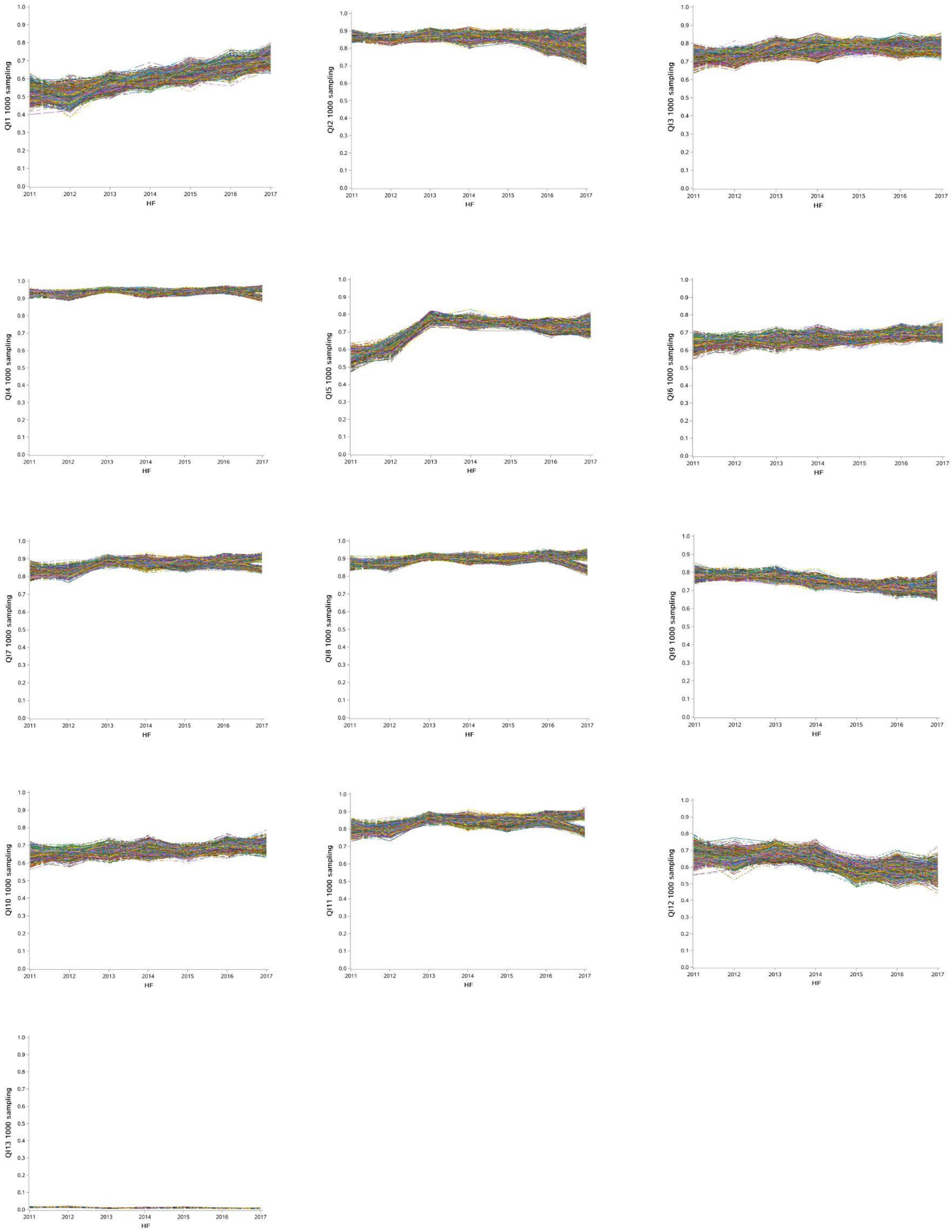

Figure S4. Trends in QIs utilization rate of HF from 2011 to 2017 by bootstrap method

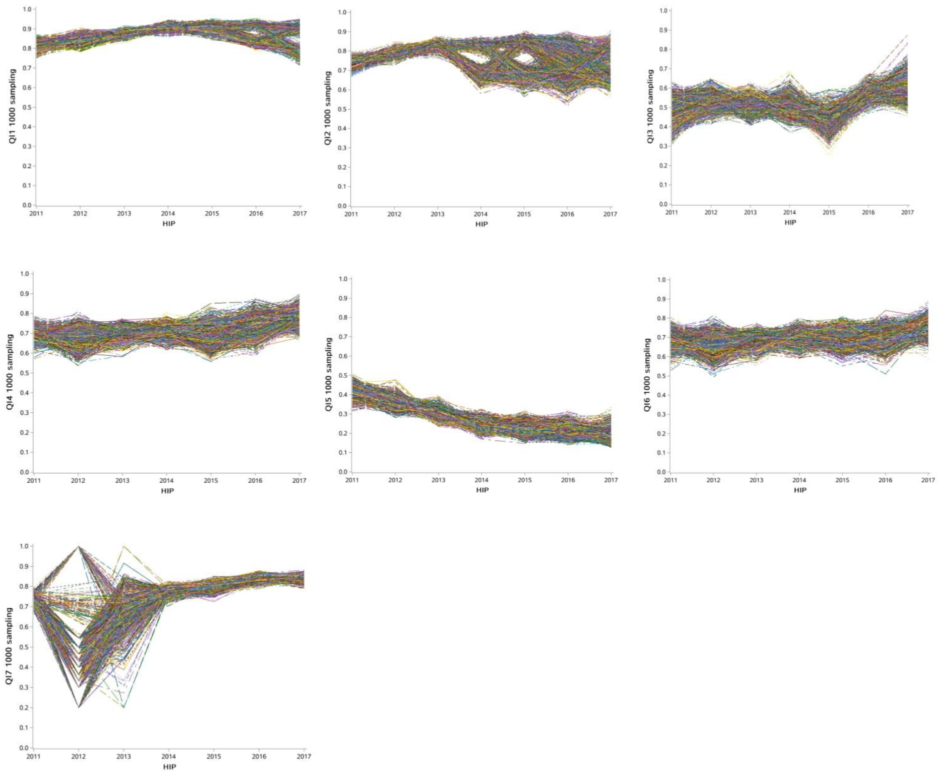

Figure S5. Trends in QIs utilization rate of H/K from 2011 to 2017 by bootstrap method

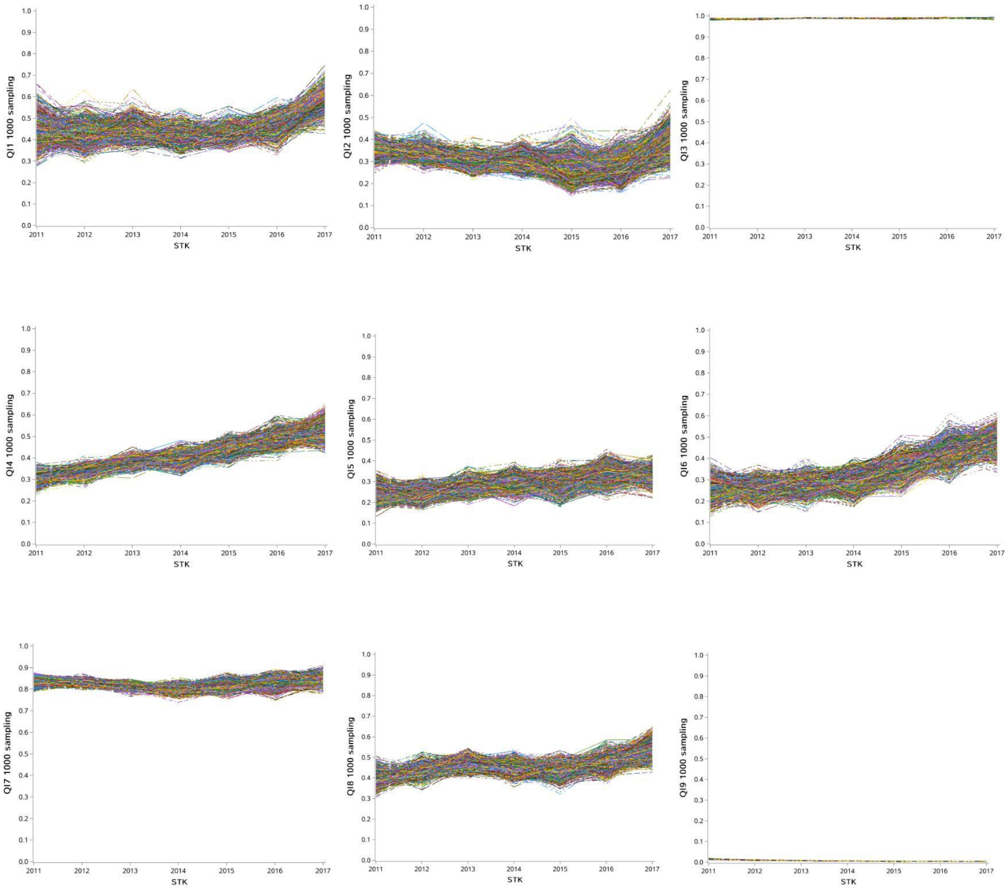

Figure S6. Trends in QIs utilization rate of AIS from 2011 to 2017 by bootstrap method

References

1. Li, X. et al. Setting performance benchmarks for stroke care delivery: Which quality indicators should be prioritized in quality improvement; an analysis in 500,331 stroke admissions. *International journal of stroke : official journal of the International Stroke Society* **16**, 727-737 (2021).
2. Meng, Q. et al. Trends in access to health services and financial protection in China between 2003 and 2011: a cross-sectional study. *Lancet (London, England)* **379**, 805-814 (2012).
3. National Bureau of Statistics of China. China Statistical Yearbook 2010. Accessed September 22, 2020. http://www.stats.gov.cn/tjsj/ndsj/2010/indexeh.htm.
